# Microbial Tracking-2, a metagenomics analysis of bacteria and fungi onboard the International Space Station

**DOI:** 10.1186/s40168-022-01293-0

**Published:** 2022-06-29

**Authors:** Camilla Urbaniak, Michael D. Morrison, James B. Thissen, Fathi Karouia, David J. Smith, Satish Mehta, Crystal Jaing, Kasthuri Venkateswaran

**Affiliations:** 1 Biotechnology and Planetary Protection Group, Jet Propulsion Laboratory, California Institute of Technology, Pasadena, CA 91109 USA; 2 Physical and Life Sciences Directorate, Lawrence Livermore National Laboratory, Livermore, CA USA; 3 KBRwyle, NASA Ames Research Center, Moffett Field, Mountain View, CA USA; 4 Department of Pharmaceutical Chemistry, University of California San Francisco, San Francisco, CA USA; 5 Blue Marble Space Institute of Science, Exobiology Branch, NASA Ames Research Center, Moffett Field, CA 94035 USA; 6 Space Biosciences Research Branch, NASA Ames Research Center, Moffett Field, Mountain View, CA USA; 7 JesTech, NASA-Johnson Space Center, Houston, TX USA

**Keywords:** International Space Station, Microbial monitoring, Microbiome, Metagenomics, Microbial tracking, Built environment

## Abstract

**Background:**

The International Space Station (ISS) is a unique and complex built environment with the ISS surface microbiome originating from crew and cargo or from life support recirculation in an almost entirely closed system. The Microbial Tracking 1 (MT-1) project was the first ISS environmental surface study to report on the metagenome profiles without using whole-genome amplification. The study surveyed the microbial communities from eight surfaces over a 14-month period. The Microbial Tracking 2 (MT-2) project aimed to continue the work of MT-1, sampling an additional four flights from the same locations, over another 14 months.

**Methods:**

Eight surfaces across the ISS were sampled with sterile wipes and processed upon return to Earth. DNA extracted from the processed samples (and controls) were treated with propidium monoazide (PMA) to detect intact/viable cells or left untreated and to detect the total DNA population (free DNA/compromised cells/intact cells/viable cells). DNA extracted from PMA-treated and untreated samples were analyzed using shotgun metagenomics. Samples were cultured for bacteria and fungi to supplement the above results.

**Results:**

*Staphylococcus* sp. and *Malassezia* sp. were the most represented bacterial and fungal species, respectively, on the ISS. Overall, the ISS surface microbiome was dominated by organisms associated with the human skin. Multi-dimensional scaling and differential abundance analysis showed significant temporal changes in the microbial population but no spatial differences. The ISS antimicrobial resistance gene profiles were however more stable over time, with no differences over the 5-year span of the MT-1 and MT-2 studies. Twenty-nine antimicrobial resistance genes were detected across all samples, with macrolide/lincosamide/streptogramin resistance being the most widespread. Metagenomic assembled genomes were reconstructed from the dataset, resulting in 82 MAGs. Functional assessment of the collective MAGs showed a propensity for amino acid utilization over carbohydrate metabolism. Co-occurrence analyses showed strong associations between bacterial and fungal genera. Culture analysis showed the microbial load to be on average 3.0 × 10^5^ cfu/m^2^

**Conclusions:**

Utilizing various metagenomics analyses and culture methods, we provided a comprehensive analysis of the ISS surface microbiome, showing microbial burden, bacterial and fungal species prevalence, changes in the microbiome, and resistome over time and space, as well as the functional capabilities and microbial interactions of this unique built microbiome. Data from this study may help to inform policies for future space missions to ensure an ISS surface microbiome that promotes astronaut health and spacecraft integrity.

Video Abstract

**Supplementary Information:**

The online version contains supplementary material available at 10.1186/s40168-022-01293-0.

## Introduction

The microbiome of the built environment has been increasingly linked to human health and disease. The composition of built environments is largely influenced by its occupants [1–4] but can also be affected by external features such as seasonal variations [5, 6], architectural design [7], and level of urbanization [8]. The International Space Station (ISS) is a unique hermetically sealed built environment and being one of the most isolated inhabited built environments; access to it is highly controlled and limited. In between the routine arrival of new supplies and crew members, the ISS is completely isolated from external microbial sources. The arrival of new crew members and supplies is therefore the only source for introducing new micro-organisms to the ISS. These isolated conditions have raised concerns about the introduction and proliferation of potentially harmful microorganisms into the microbial communities of piloted spaceflight and how this could affect human health.

For decades, space-faring nations were interested in the influence of microgravity, radiation, and other spaceflight conditions on the survival and proliferation of microorganisms. During the Apollo program, NASA collected pre- and post-flight samples from locations within the Command Module as part of a program to identify medically relevant microbes and to elucidate changes in the crew microflora [9]. Subsequently, Skylab surfaces were swabbed pre-launch and periodically throughout the three manned missions to characterize the bacterial exchange between the crew and internal surfaces [10]. The Soviet Union regularly monitored the microflora of Salyut 6, 7, and Mir during their operation. Onboard station Mir, air samples were collected approximately once a month, and surface samples from various locations throughout the spacecraft were collected at the end of each mission [11–13]. Thorough monitoring onboard Mir revealed potential health and equipment risks from the bacteria and fungi that colonized the interior surfaces [14]. These culture-based surveillance procedures were instrumental in developing the microbial surveillance standards for the ISS [15, 16].

Since inception, to maintain the cleanliness of ISS, traditional culture-based approaches are being used and microbial burden measured. However, research activities are being conducted to characterize the microbiome of ISS surfaces and air using state-of-the-art molecular techniques. Air, water, and surface samples were collected during the initial stage of habitation of the ISS to identify the baseline microbiota [17]. Since then, several studies have investigated the microbial population of ISS dust [18–20], surfaces [21, 22], and water [23]. In addition to cataloging the microbial population of the ISS, studies have also focused on the persistence of antimicrobial resistance (AMR) genes [24] and pathogens [25] onboard the ISS. These investigations provide important information for the long-term health and safety of crew members.

The previous Microbial Tracking-1 (MT-1) study [26] examined the community structure and function of eight ISS surfaces over a period of 14 months, during three sampling sessions. The Microbial Tracking-2 (MT-2) study continued this objective and further sampled the same surface locations over the course of 1.5 years, during four additional flight sessions. In this paper, the MT-2 samples were analyzed for species presence and stability of the entire microbial community. The microbial diversity and composition for each location and time point were characterized to reveal how the microbial population was changing over time. The persistence of antimicrobial resistance genes was also analyzed to evaluate the stability of the ISS resistome in a changing microbiome. Co-occurrence analysis between bacterial and fungal species was analyzed to gain insight into the microbial associations in this unique built environment. Genomes were assembled from the metagenome reads (referred to as metagenome-assembled genomes, or MAGs) with taxonomy and functional assessment analyses performed on these MAGs. This paper also compared the results obtained during the previous MT-1 study. Together, these studies provide snapshots of the microbial population on the ISS across 5 years, allowing for dynamics and stability patterns of the surface microbiome to be evaluated.

## Results

Thirty-two surface samples were collected over the course of 14 months (2017–2018) from the ISS. The locations sampled are listed in Table 1 and correspond to the same eight locations that were sampled during the MT-1 study that ran from 2015 to 2016 [26]. In addition to the sampling wipes, wipes that were taken out of the kits and exposed to the ISS environment (but not used for sampling) were designated as controls and processed alongside the samples. The flight sampling sessions are denoted as flights 4–7 (F4–F7), while the flight sampling sessions for the previously published MT-1 study are denoted as flights 1–3 (F1–F3).

**Table 1:** Locations sampled across the International Space Station. All eight 8 locations were sampled over the course of four different flights—flight 4 (F4), flight 5 (F5), flight 6 (F6), and flight 7 (F7). Flight 4 was sampled in 2017, and F5, F6, and F7 were sampled throughout 2018

Location number	Location description	ISS module
L1	Port panel next to the cupola	Node 3
L2	Waste and hygiene compartment	Node 3
L3	Advanced resistive exercise device (ARED) foot platform	Node 3
L4	Dining table	Node 1
L5	Overhead 4	Node 1
L6	Permanent multipurpose module (PMM) Port 1	PMM
L7	Lab 3 overhead	LAB
L8	Port crew quarters, bump out exterior aft wall	Node 2

### Microbial burden

All microbial domains of life were detected on the ISS, with bacteria being the most represented (82%), followed by fungi (10%), viruses (0.06%), and archaea (0.03%), along with reads that could not be identified (7%) (Dataset S1). Of the total population, 38% could be attributed to viable organisms, based on the read counts obtained from propidium monoazide (PMA)-treated samples (which measures viable/intact organisms), with bacteria still being the most dominant group [bacteria (58%), fungi (37%), viruses (0.01%), archaea (0.004%), unclassified (4%)] (Dataset S1).

**Fig. 1:**
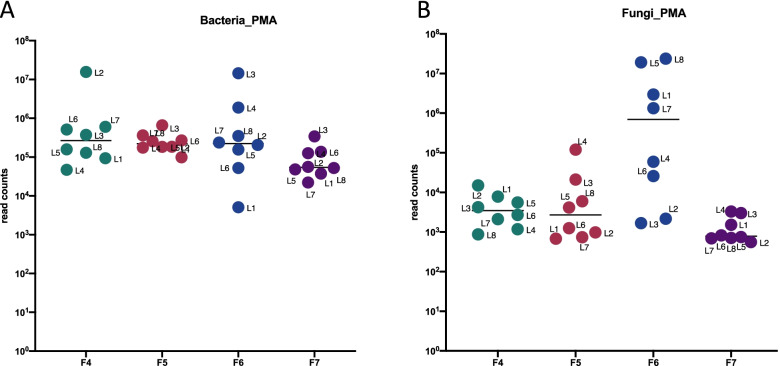
Metagenomic read counts obtained from surface wipes sampled during flights 4–7. Each dot of the graph represents a location sampled on the ISS. The line represents the average read count for a specific flight from all eight locations. PMA-treated samples were analyzed which measures viable/intact cells. **A** Read counts of the bacterial population. **B** Read counts of the fungal population. The Kruskal–Wallis one-way analysis of variance test, followed by the post hoc false discovery rate was used to compare the read counts between flights

The viable bacterial and fungal load was compared between F4 to F7. The average bacterial read count across all four flights was 7 × 10^5^ counts/m^2^, with no statistically significant differences between them (Fig. 1A). This contrasts with the fungal read counts which did differ between flights, with F6 having the highest average sequence reads at 7.0 × 10^5^ counts/m^2^ (Fig. 1B).

Plate count assays were also performed. The cultivable bacterial burden was highest at F6, with an average of 2.7 × 10^6^ cfu/m^2^, and lowest at F7, with 1.0 × 10^2^ cfu/m^2^ (Fig. S1A). F6 also had the highest cultivable fungal burden at 1.1 × 10^4^ cfu/m^2^, with F7 having below detectable fungal counts (Fig. S1A).

### Microbial diversity

#### Alpha diversity

**Fig. 2:**
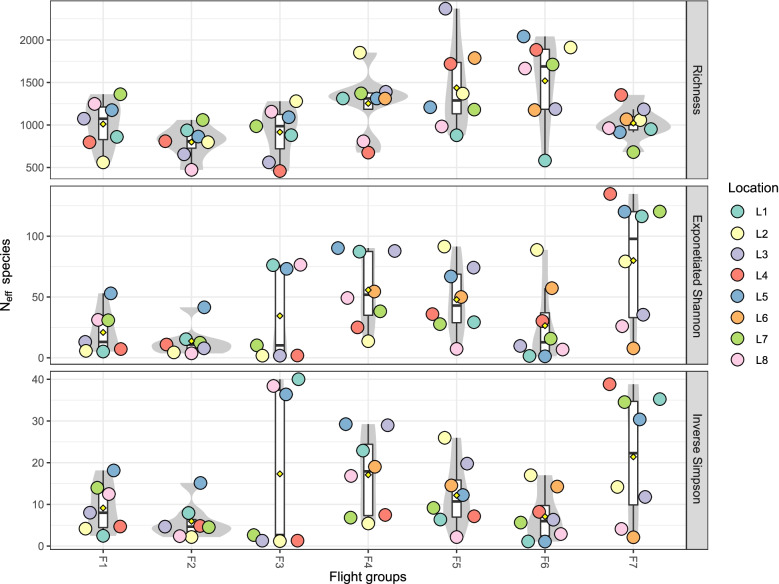
Alpha diversity metrics for MT-1 and MT-2 samples. The species richness (top row), exponentiated Shannon index (middle row), and inverse Simpson index (bottom row) are shown for each sample. Samples are grouped by flight group and colored by surface location

Three alpha diversity indices were used to measure the microbial diversity within each sample: observed richness (Fig. 2, top panel), exponentiated Shannon index (Fig. 2, middle), and reciprocal Simpson index (Fig. 2, bottom). Included for comparison were also the MT-1 samples from F1–F3. The Kruskal-Wallis statistical test was used to determine the differences between flights, and no significant differences were observed across any of the seven flights. There were also no differences across any of the eight locations sampled (Fig. S2).

#### Beta diversity

**Fig. 3:**
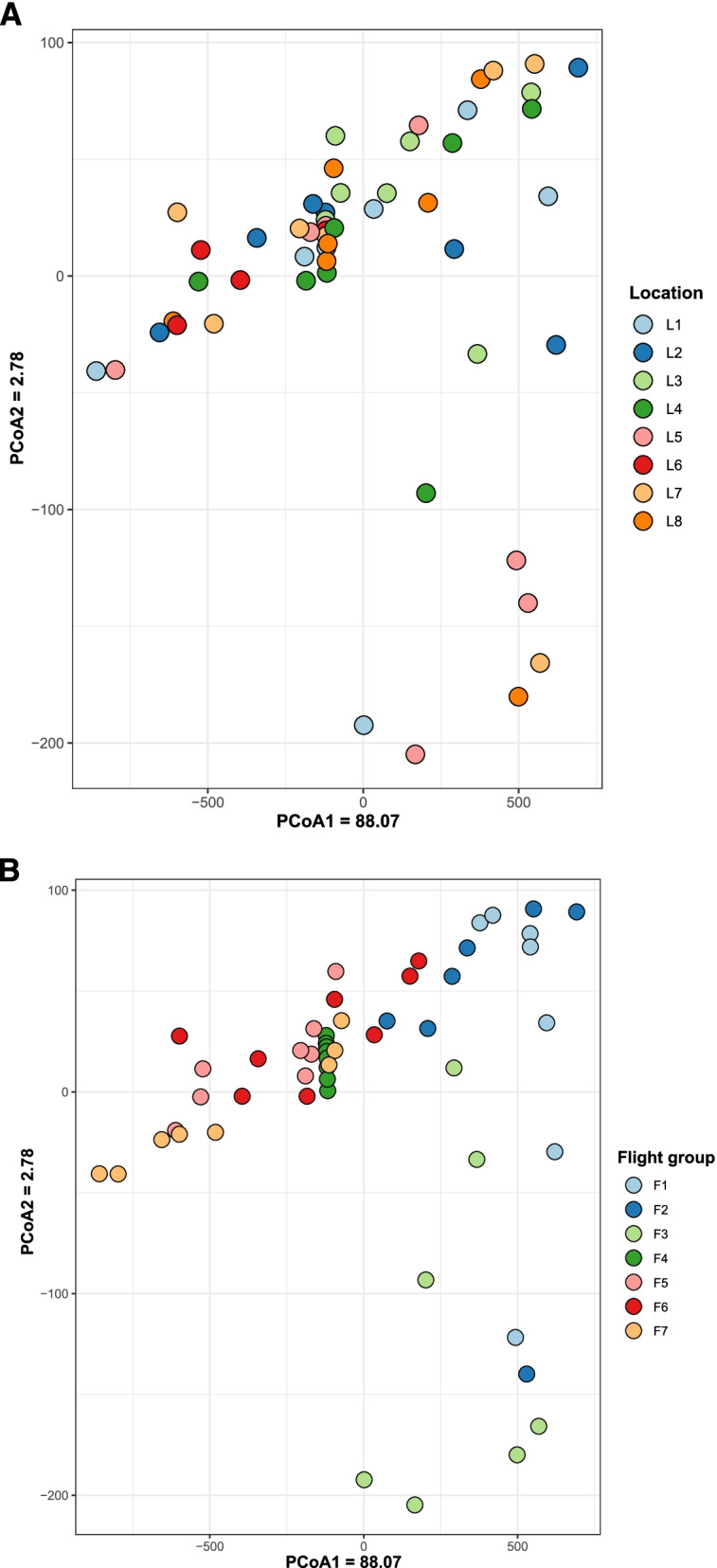
PCoA plot of MT-1 and MT-2 surface samples. Samples were colored by surface location (**A**) and flight group (**B**) to visualize the sample clustering. The distance between the samples was determined using the Euclidean distance

Comparison between surface samples was visualized using principal coordinates analysis (PCoA) using Euclidean distances (Fig. 3) and organized by location (Fig. 3A) and flight (Fig. 3B) to identify possible trends. No apparent microbiome differences were observed between locations; however, samples did appear to cluster by flight group. These observations were supported by PERMANOVA analysis of the Euclidean distances which found a significant difference between centroids and dispersions of the flight groups (*P* < 0.001) but not between the locations (*P* = 0.229). Surface samples were also visualized and analyzed by non-metric multidimensional scaling (NMDS) using Jaccard distances (Fig. S3), and the same trends were noted as with the PCoA data above.

#### Taxonomic profile of ISS microbial community

**Fig. 4:**
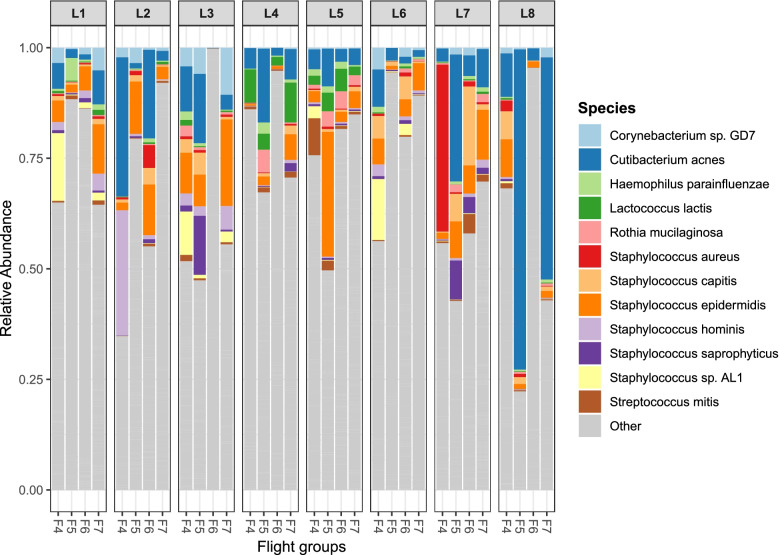
Top 12 most abundant bacterial species across all surface samples. The percent of mapped reads for each species in each sample. Samples were grouped in columns by surface location and arranged in order by flight group. “Other” refers to those bacterial species detected that were not in the top 12

The top 12 most abundant bacterial species across all surface samples are shown in Fig. 4 with all but one being associated with the human microbiome. *Propionibacterium acnes* and the six *Staphylococcus* spp.: *Staphylococcus aureus*, *Staphylococcus capitis*, *Staphylococcus epidermidis*, *Staphylococcus hominis*, *Staphylococcus saprophyticus*, and *Staphylococcus* sp. AL1, reside on the skin and mucous membranes of humans. *Haemophilus parainfluenzae*, *Rothia mucilaginosa*, and *Streptococcus mitis* are part of the normal oral microbiome. *Corynebacterium* sp. GD7 (reclassified as *Corynebacterium ihumii*) was first isolated from a fecal sample. *Lactococcus lactis* is used in the dairy industry for the production of various cheeses and buttermilk. The read counts for the top 12 bacteria, organized by flight and location (including controls), are shown in Fig. S4.

**Fig. 5:**
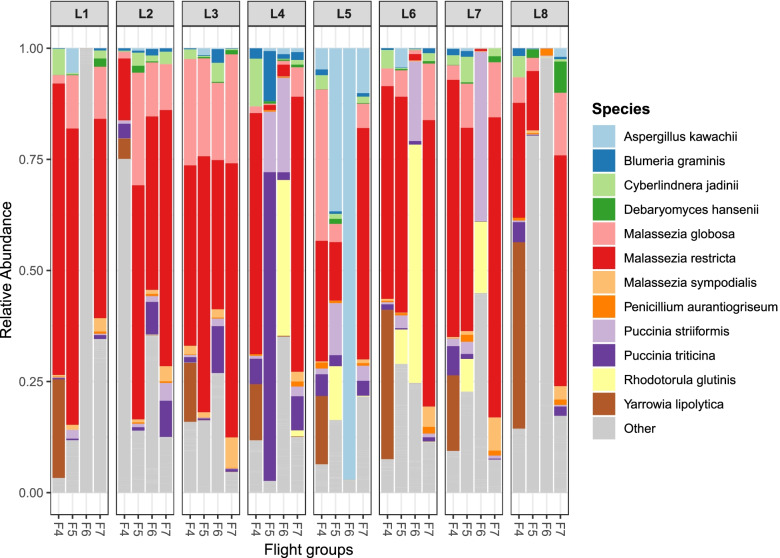
Top 12 most abundant fungal species across all surface samples. The percent of mapped reads for each species in each sample. Samples were grouped in columns by surface location and arranged in order by flight group. “Other” refers to those fungal species detected that were not in the top 12

The top 12 most abundant fungal species across all surface samples are shown in Fig. 5. These include *Malassezia globose*, *Malassezia restricta*, and *Malassezia sympodialis* which are part of the healthy skin microflora. The *Malassezia* species constitute the majority of fungal reads in most surface samples regardless of location. *Rhodotorula glutinis* and *Yarrowia lipolytica* are common environmental fungi, and *Aspergillus kawachii*, *Cyberlindnera jadinii*, and *Debaryomyces hansenii* are used in the food industry. *Blumeria graminis* (barley powdery mildew), *Penicillium aurantiogriseum* (asparagus and strawberry pathogen), *Puccinia striiformis* (Stripe rust), and *Puccinia triticina* (wheat leaf rust) are all associated with plant diseases but pose no known harm to human health. The read counts for the top 12 fungi, organized by flight and location (including controls), are shown in Fig. S5.

Differential abundance analysis of taxa was performed at the species level. Samples were grouped by flight group (i.e., F4, F5, F6, and F7) or location (i.e., dining table, crew quarters, etc.) for analysis. While there were no differentially abundant species across locations (*P* < 0.05), there were 20 differentially abundant species across flights (*P* < 0.05) (Table 2).

**Table 2:** Species identified as differentially abundant between the sampling flight groups. Kruskal-Wallis (KW) and general linear model (GLM) raw *P* values and Benjamini and Hochberg adjusted *P* values (*Q* values) are shown for each species

Species	***P*** value (KW)	Q value (KW)	***P*** value (GLM)	Q value (GLM)
*Thiobacillus thioparus*	1.36E−04	2.11E−01	9.60E−09	1.17E−05
*Magnetospirillum magnetotacticum*	2.31E−04	2.13E−01	1.98E−08	3.01E−05
*Yarrowia lipolytica*	1.70E−04	2.09E−01	8.49E−07	4.04E−04
*Tetragenococcus halophilus*	1.79E−04	2.11E−01	8.25E−07	7.28E−04
*Ralstonia pickettii*	3.87E−04	2.20E−01	5.01E−06	1.31E−03
*Methyloversatilis universalis*	7.02E−04	2.36E−01	1.81E−05	5.07E−03
*Acidovorax* sp. *CF316*	4.65E−04	2.25E−01	1.74E−05	5.47E−03
*Lactococcus raffinolactis*	7.31E−03	4.10E−01	4.83E−05	1.22E−02
*Thiobacillus denitrificans*	4.39E−04	2.22E−01	1.54E−04	1.43E−02
*Nevskia ramosa*	2.04E−04	2.13E−01	1.14E−04	1.49E−02
*Leuconostoc lactis*	8.67E−04	2.45E−01	1.26E−04	2.04E−02
*Sphingobium* sp. *Ant17*	8.20E−04	2.47E−01	3.02E−04	2.55E−02
*Leuconostoc mesenteroides*	2.65E−03	3.25E−01	1.79E−04	2.60E−02
*Cellvibrio* sp. *BR*	1.04E−03	2.54E−01	5.15E−04	3.29E−02
*Bradyrhizobium japonicum*	5.11E−03	3.85E−01	3.15E−04	3.53E−02
*Emiliania huxleyi*	2.54E−03	3.12E−01	3.86E−04	4.08E−02
*Herbaspirillum huttiense*	1.27E−03	2.64E−01	7.49E−04	4.27E−02
*Bacillus subtilis*	1.85E−03	2.99E−01	7.69E−04	4.39E−02
*Pectobacterium carotovorum*	4.04E−03	3.45E−01	4.60E−04	4.64E−02
*Chloroflexi bacterium JGI 0002000-F10*	7.12E−04	2.36E−01	5.80E−04	4.79E−02

### Functional analyses

#### Co-occurrence of microbial taxa

**Fig. 6:**
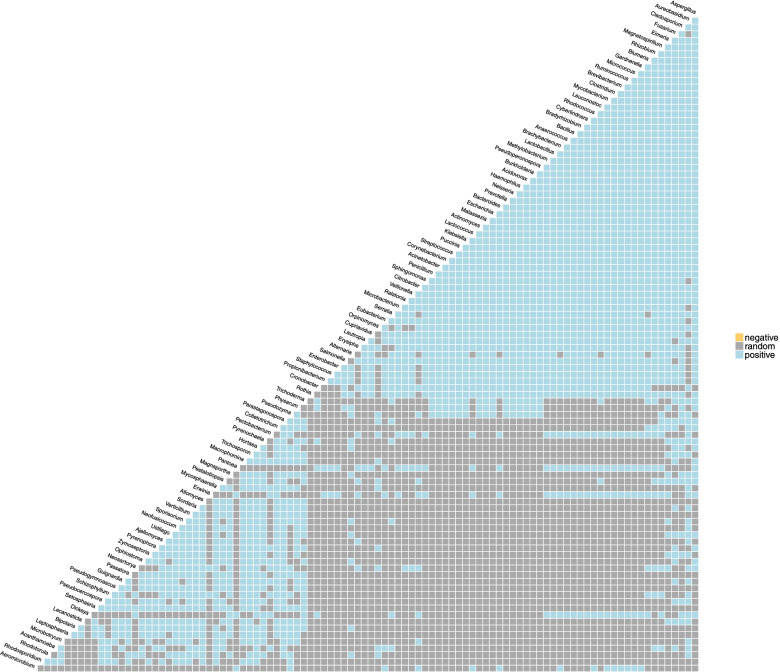
Co-occurrence analysis. The co-occurrence analysis was performed using the 50 most abundant genera to determine the associations among organisms. The matrix shows which genera co-occurred with each other. Blue shows the positive co-occurrences (i.e., if you find one in the community, it is likely you will find another—for example, *Aspergillus* and *Fusarium*), yellow shows the negative co-occurrences (i.e., if you find one, it is likely the other will be absent), and gray shows the random occurrences that are most likely due to random chance alone—for example, *Cladosporium* and *Fusarium*

Inference of microbial ecological interactions was obtained from patterns of occurrence of the genera present in the samples. PMA-treated samples were analyzed to determine which viable bacterial and fungal genera were found to co-associate with each other. The top 50 most abundant bacteria and fungi were included in the analysis. As seen from the genus association profile in Fig. S6, *Aspergillus* had the most co-associations, with other fungi and bacteria while *Pseudomonas* did not have any. The gene association matrix in Fig. 6 shows which bacteria and fungi co-occurred with each other. Of the bacteria and fungi that were included in the analysis, the associations were all positive (i.e., presence of one likely means the presence of another) with no negative associations (i.e., presence of one likely means the absence of another).

#### Antimicrobial resistance profiles

Twenty-nine AMR categories were detected in the metagenomics dataset with multidrug and macrolide/lincosamide/streptogramin (MLS) resistance having the highest read counts and found in all 32 samples (Fig. S7). Bacitracin, beta-lactam, diaminopyrimidine, and tetracycline resistance were also found across all samples, except from F6_1S (surface location # 1 [cupola], F6; Fig. S7). The “unclassified” category was also found in all samples (except F6_1S), and the genes that were grouped into this category were all DNA-binding regulatory proteins involved in the stress response.

**Fig. 7:**
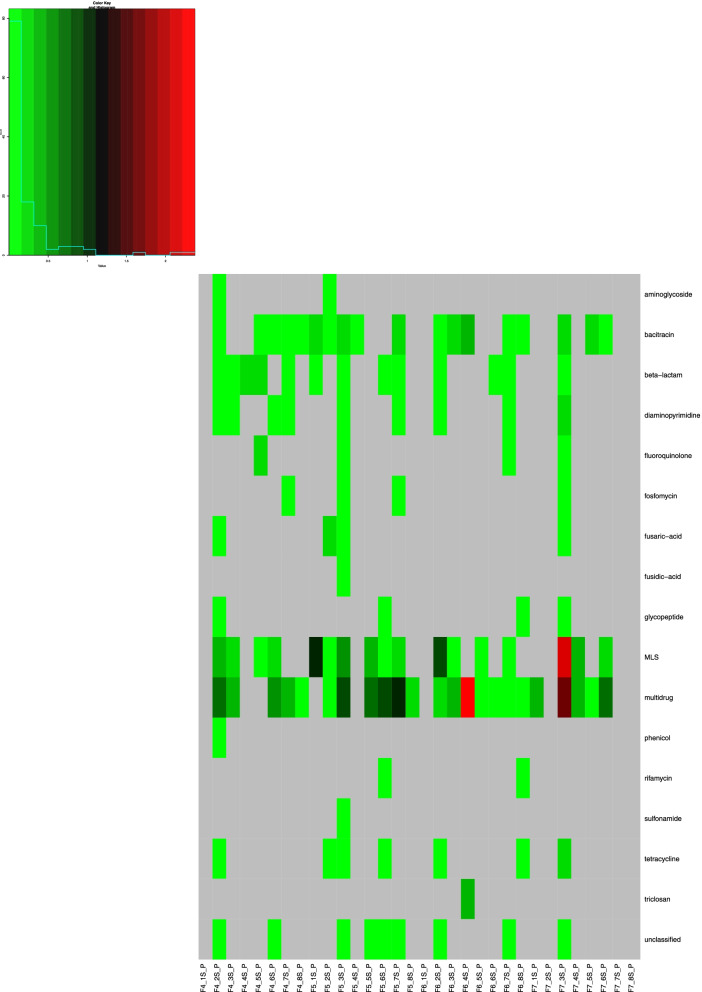
Heat map of the ISS viable resistome. The metagenomic dataset was analyzed with DeepARG to look for anti-microbial resistance genes. The genes were then grouped into classes of resistance (i.e., “beta-lactam”), which is displayed in this heatmap (*y*-axis). The heatmap shows the relative abundance of each resistance class in each sample collected, with red being the most abundant and green the least. Gray indicates zero counts detected in that sample. The samples analyzed (*x*-axis) are from the PMA-treated samples which detect viable/intact cells. *x*-axis naming: Fx_xS_P, with F referring to the flight (i.e., F4) and S referring to the location of the surface sampled (i.e., 1S is location 1, the port panel next to the cupola). The AMR counts were normalized based on the 16S rRNA counts

Of the 29 AMR categories detected above, 17 were detected in the PMA-treated group. Multidrug and MLS resistance genes still had the highest counts, though were not found in every sample, as in the non-PMA group (Fig. 7).

**Fig. 8:**
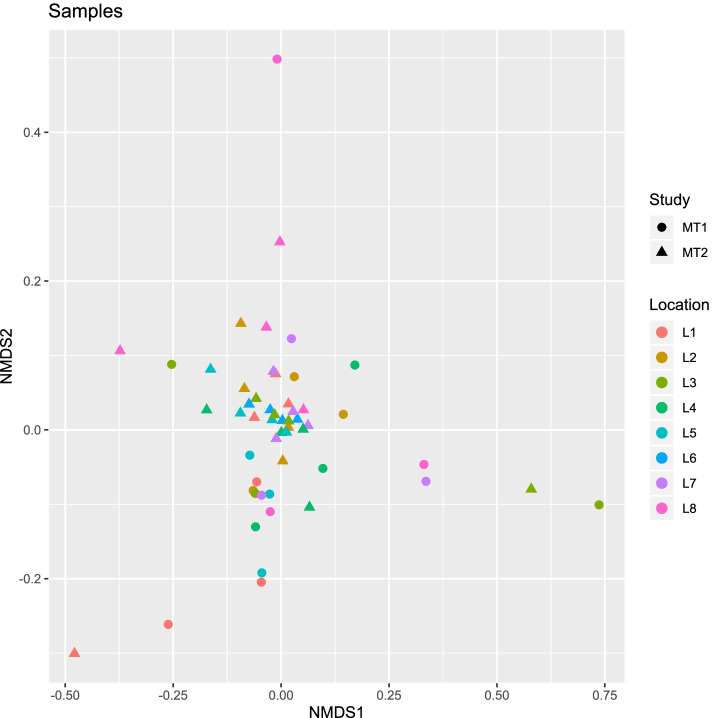
Comparison of ISS viable resistome among flights. NMDS plot comparing the antimicrobial resistance profile of the ISS community. Metagenomic data collected from the PMA-treated samples (viable/intact cells) during each of the flight sampling sessions for both the current MT-2 study (F4–F7) and the previous MT-1 study (F1–F3) was analyzed by DeepARG. Each dot represents a sample and takes into account the presence of an antimicrobial resistance gene and its abundance. Locations are depicted by colors and MT-1 vs MT-2 study, by symbols

Multi-dimensional scaling analysis comparing MT-2 and MT-1 samples (both PMA and non-PMA treated) showed no differences in the AMR profiles between the two studies, suggesting that the ISS resistome is relatively stable (Fig. 8).

#### Metagenome-assembled genomes

**Fig. 9:**
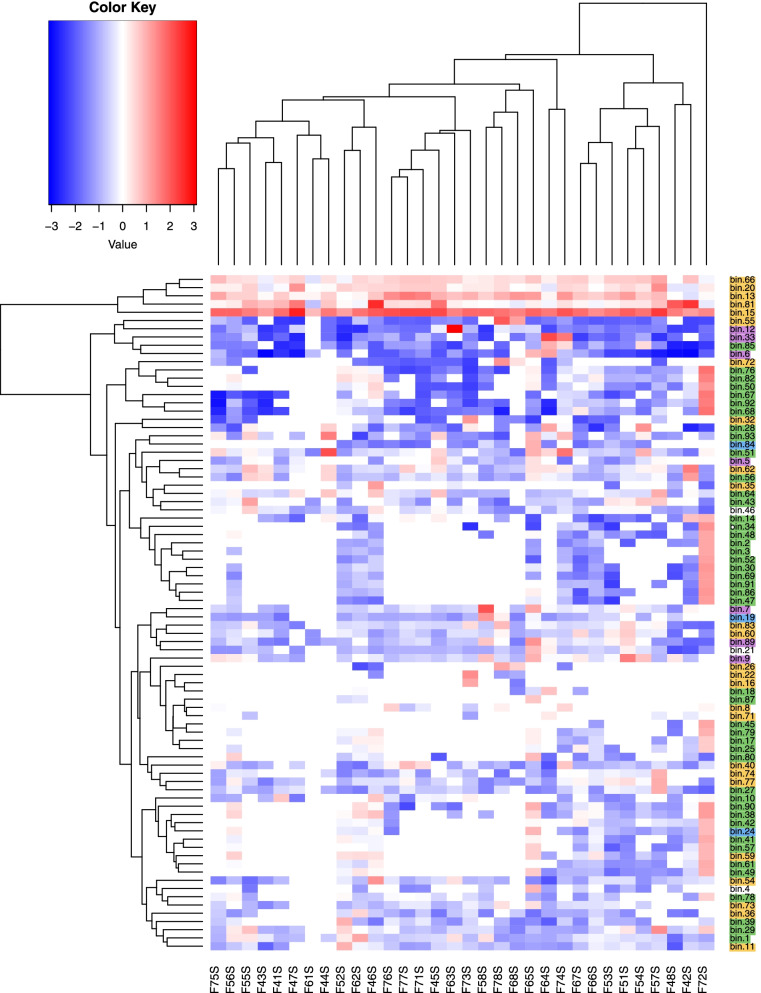
MAG abundance heatmap. Genomes were assembled from the metagenomics reads and placed into “bins,” with each “bin” representing one metagenomic assembled genome (“MAG”). These bins were generated from the metagenomic data consisting of the 32 samples collected during the course of the study. Each bin is shown on the *y*-axis. The abundances are expressed as genome copies per million reads and presented, with the log10 values plotted. Red represents a high relative abundance and blue a low relative abundance. The white color represents the absence of that bin in the sample. The bin numbers were highlighted based on the phylum it was assigned to, with yellow = Actinobacteria, green = Firmicutes, blue = Bacteroidetes, and purple = Proteobacteria. Full taxonomic info for each bin can be found in Dataset S2. The *x*-axis shows the sample that was analyzed and the relative abundance of that bin (i.e., MAG) in that sample. *x*-axis naming: Fx_xS, with F referring to the flight (i.e., F4) and S referring to the location of the surface sampled (i.e., 1S is location 1, the port panel next to the cupola)

Metagenomic-assembled genomes (MAGs) were constructed from the 32 samples collected during flights 4–7, with 82 MAGs able to be assembled using a 50% minimum completion cutoff. Of those 82, 15 had over 90% completion. The percent completion, contamination, GC content, taxonomy, N50 (a measure of assembly quality), and genome size are summarized in Dataset S2. The heatmap in Fig. 9 shows the distribution and abundance of each MAG across all samples.

**Fig. 10:**
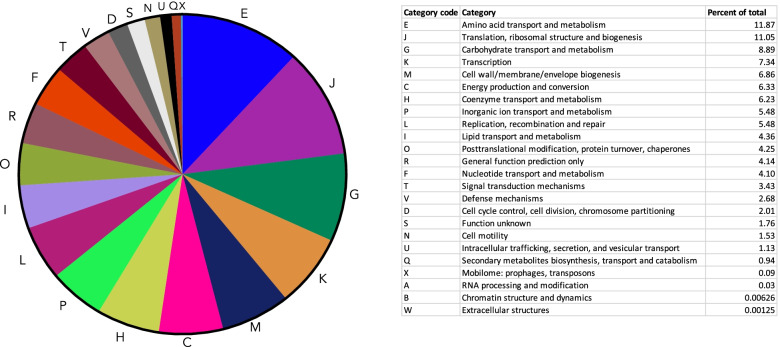
Pie chart of the cluster of orthologous groups. Functional assessment was performed by comparing the annotated MAGs against the COG database and assigning each COG ID to a category. The counts from each category are displayed as a percentage of the total counts in the pie chart

To extract functional information from these MAGs, annotation was performed on each MAG. Clusters of orthologous groups (COG) information was extracted from each annotated MAG and compiled together to provide a functional assessment of the collective microbial community, with the complete list of each COG ID presented in Dataset S3. Each annotated gene with a COG ID was then grouped together based on COG functional categories and the proportion of each functional category represented in the pie chart in Fig. 10. The most represented functional groups were amino acid transport and metabolism (12%); translation, ribosomal structure, and biogenesis (11%); and carbohydrate transport and metabolism (9%). Genes/proteins involved in the defense mechanisms and the exchange of genetic material (i.e., mobilome) had a relatively small representation (3% and 0.1%, respectively).

We decided to examine MAG *Pseudomonas granadensis* (bin.33, 99.38% completion) in further detail because it was 99.97% similar (compared by average nucleotide identity) to the whole-genome sequences of *P. granadensis* isolates, cultured during this study (flight 4, dining table) [27]. In addition, *P. granadensis* belongs to the *P. fluorescens* group which has many biologically relevant properties. While generally considered a BSL-1 organism, it can cause severe diseases in people who are immunocompromised and can lead to the spoilage of milk. It also exhibits biocontrol properties through its protection of plant roots from plant pathogens. Examination of MAG *P. granadensis* (bin.33) showed 17 genomic islands, which is indicative of horizontal gene transfer (HGT) events. Functional analysis showed that 3% of its genome was made up of virulence and defense genes specifically related to tolerance/resistance of fluroquinolones, beta-lactams, copper, cobalt-zinc-cadmium, streptothricin, chromium, and colicin E2, as well as having multi-drug efflux pumps (Fig. S8). The genome also carried genes that could increase survival under unfavorable conditions, such as those involved in biofilm formation; osmotic, periplasmic, and oxidative stress; and dormancy and sporulation (Fig. S8).

## Discussion

The microbiome of eight surfaces from across the ISS was sampled during four separate sampling sessions (F4–F7). The viable microbiome was assessed with shotgun metagenomic sequencing of samples that had been treated with PMA, as well as with plate count assays. Both techniques were consistent in their results, showing that the microbiome was similar across all eight locations for a given flight and validates what was previously published for MT-1 [26, 28] and with other ISS surface microbiome studies [29]. The spatial uniformity in this hermetically sealed, closed habitat suggests that microbial movement occurs readily, which should be taken into consideration if a situation arises that requires microbial sequestration.

While no spatial differences were found, temporal differences did exist, with different microbial profiles and microbial loads observed among the various flights, consistent with our previous analyses [26, 28]. One influence driving these temporal differences could be the different cohorts of astronauts onboard the ISS during each sampling session. Two recent studies have noted that the crew microbiome may shape microbial composition on ISS habitable surfaces [29, 30], and in this study, the majority of bacteria and fungi that were detected from surfaces were human-associated, such as *Staphylococcus*, *Propionibacterium*, *Streptococcus*, *Haemophilus*, and *Malassezia* or those related to food products that are ingested by humans, such as *Aspergillus kawachii* and *Lactococcus lactis*. Astronaut-ISS surface exchange would parallel what has been found with Earth-based built environment studies, where human occupation significantly impacts a given indoor environment [2, 31, 32]. To garner a better understanding of just how significant human-environment interactions are in shaping the microbiomes of the ISS and the astronauts residing there, ongoing analyses by the MT-2 project team are examining astronaut data at pre-flight, in-flight, and post-flight time points and examining their microbiomes in conjunction with the ISS surface microbiome.

*Staphylococcus* sp. and *Malassezia* sp. were the most represented bacterial and fungal species, respectively, on the ISS. While bacteria dominated the ISS microbiome in both the PMA and non-PMA samples, it is worth noting that the proportion of fungi increased from 10% in the total population (viable/dead) to 37% in the viable population, while bacteria decreased from 82% (total) to 58% (viable). This would suggest that viable/intact fungal cells are more readily captured when ISS surfaces are sampled, compared to bacteria. This could be because these fungi are able to withstand the routine cleaning regimes on the ISS or the conditions surrounding the sample storage/descent to Earth/sample processing, better than those bacteria.

In addition to examining which microbes were present, we also examined their properties, which may help inform policies and practices to mitigate harmful effects on astronauts and the spacecraft. One such functional examination was that of the ISS “viable resistome” which is the composition of antimicrobial resistance genes within the PMA-treated metagenomic dataset. We found a genetic composition that could provide resistance against 17 classes of drugs, many of which were broad-spectrum antibiotics, such as aminoglycosides, beta-lactams, fluoroquinolones, and tetracycline, all of which are part of the medical toolkit onboard the ISS. The most prevalent resistance genes were against macrolide, lincosamide, and streptogramin (MLS) antibiotics and those involved in multidrug resistance, such as outer membrane porins, efflux pumps, and ABC transporters. MLS antibiotics occupy one of the leading positions among antibiotics used in outpatient treatment [33] and are used to treat a range of bacterial infections, especially methicillin-resistant staphylococcal skin and soft tissue infections [34, 35], thus making it a very important drug on the ISS. Genetic resistance to MLS was found in every single sample in the total metagenomic dataset (non-PMA-treated), and in 17 out of 32 PMA-treated samples, making MLS resistance widespread across the ISS.

The AMR resistance profiles did not differ across all seven flights, suggesting that even amidst changes in community composition (which we observed among the seven flights), the resistome of this microbial community is relatively stable and alludes to genetic redundancy. Functional redundancy has been documented in other microbial systems such as in plants [36], human gut [37], oceans [38], and soil [39]. One study has shown that HGT plays a substantial role in maintaining redundancy, and when HGT rates were high, the functional profile of the microbiome was very stable and recalcitrant to change, even when taxonomy was variable [40]. Our results emphasize the importance of deciphering microbial behavior and community interactions for better approaches to safety and health, since removing or preventing the growth of one or two species may not ameliorate any negative functional effects.

Strong microbial interactions were observed in this study through a co-occurrence analysis used to predict the likelihood of genera to co-inhabit a particular niche, either through positive associations or through negative ones. While many positive associations were calculated, no negative associations were accounted for. The genus that exhibited the most positive associations was *Aspergillus* and co-occurred with both bacteria and fungi, while at the other “end” of the spectrum, *Pseudomonas* had no associations and exhibited only random occurrences. While wet lab studies would need to be conducted to make an accurate assessment of the impact of these ISS polymicrobial community interactions on the surrounding environment and astronaut health, some inferences can be made from the literature. In our study, *Aspergillus* and *Fusarium* formed a strong association. Co-infection studies of *A. flavus* with *F. graminearum* or *F. verticillioides* in maize have shown that the presence of these *Fusarium* species leads to enhanced aflatoxin B1 (AFB1) production by *A. flavus*, even in the absence of any changes in growth, probably due to a stress response caused by fungal competition [41, 42]. AFB1 is a secondary metabolite produced by *A. flavus* and *A. parasiticus* and is a very potent carcinogen that can cause hepatocellular carcinoma in humans when ingested or when it permeates the skin [43]. While reads for *A. flavus* were extremely low on ISS surfaces, the species is naturally found in foods, such as grains, cereal, corn, and peanuts. In light of the co-occurrence interactions described above and the known fact that the ISS is a stressed environment for microbes, which could increase AFB1 production as well as other mycotoxins, it may be necessary to pay close attention to microbial monitoring of food sources destined for space.

*Aspergillus* and *Staphylococcus* were another positive association found in the dataset and may prove to be beneficial for both crew and the ISS. In an in vitro study analyzing mixed *A. fumigatus*-*S. aureus* biofilms, it was shown that *S. aureus* strongly inhibited the development of *A. fumigatus* biofilms [44]. The antibiofilm effects of *S. aureus* on *A. fumigatus* were a lack of extracellular matrix (ECM) disorganized fungal structures, abortive hyphae, and limited hyphal growth [44]. Conidia were also scarce, had modifications on their surface, and showed lysis [44]. In contrast, *A. fumigatus* single culture biofilms showed extremely organized structures, abundant hyphal growth, hyphal anastomosis, and channels, as well as some conidia and ECM [44]. *Aspergillus* biofilms (i) cause aspergillosis, an infection causing allergic reactions, lung disease, and infections in other organs [45, 46]; (ii) have been implicated in microbially induced corrosion of bronze and copper [47]; and (iii) have been found in tap water in private homes, hospitals, and industrial premises, resulting in altered taste, odor, the production of allergenic compounds, and mycotoxins [48, 49]. *Aspergillus* biofilms are difficult to eradicate once formed, so preventing their formation, possibly with mixed microbial communities of the “right” organisms, may be the best way to combat its effects on the ISS.

Functional assessment was also performed by assembling reads into contigs to obtain metagenome-associated genomes or “MAGs.” Across the samples collected, 82 MAGs were able to be constructed and annotated with 15 having over 90% completeness, and comprising environmental, human-associated, and probiotic bacteria. The functional profile of the collective MAGs showed the highest representation of (i) amino acid transport and metabolism and (ii) translation, ribosomal structure, and biogenesis. While these two pathways are among the main metabolic pathways in bacteria and fungi, their relative abundances were higher than carbohydrate utilization pathways. This may be significant in the context of a recent study comparing “unrestricted” built environments (public houses, public buildings, private houses) with “confined” built environments (intensive care units (ICU), cleanrooms, cleanroom gowning areas) where it was shown that each of the three “confined” environments had a considerably higher representation of amino acid utilization compared to carbohydrate utilization, with the opposite being true for the “unrestricted” environments (higher carbohydrate utilization compared to amino acids) [50]. While there are too few comparative studies to make any assumptions, it does raise the question as to whether organisms in more extreme built environments such as the ISS, ICU, and cleanrooms rely on different metabolic processes to survive and/or proliferate. The ability to generate good-quality MAGs from this dataset will allow us to better understand the dynamics of exchange across the ISS. For example, future analyses by the MT-2 project team will compare the ISS surface MAGs and astronaut MAGs (who were onboard the ISS at the time of collection) to track the movement of strains across ISS locations and across astronauts. Questions we hope to address in the forthcoming work are what factors contribute to the spread of certain organisms from one location to another, how long does a strain persist once deposited on a surface and after cleaning, and whether there is an exchange between astronauts even if they were not on board at the same time.

## Conclusion

Utilizing various metagenomics analyses as well as culture, this paper has provided a comprehensive analysis of the ISS surface microbiome, showing microbial burden, bacterial and fungal species prevalence, how the microbiome and resistome changes over time and space, and the functional capabilities and microbial interactions of this unique built environment. Data from this study may help to inform policies for future space missions to ensure an ISS surface microbiome that promotes astronaut health and spacecraft integrity.

## Methods

### Sample kit preparation and sample collection

Sampling wipes were prepared at the Jet Propulsion Laboratory (JPL; Pasadena, CA) by moistening polyester wipes (9″ × 9″; ITW Texwipe, Mahwah, NJ), each with 15 mL of sterile molecular grade water (Sigma-Aldrich, St. Louis, MO). Moistened wipes were aseptically transferred to a sterile zip lock bag and then assembled into the ISS sampling kit (at NASA Ames Research Center (ARC)), which also contained sterile gloves (KIMTEC Pure G3 White; Nitrile Clean-room Certified; Cat. HC61190), benzalkonium chloride wipes (Hygea®, PDI-BZK D35185), and sterile bitran bags (VWR, Cat. 4742-S) for return. The implementation team at NASA ARC delivered the kit to the Cargo Mission Contract at Johnson Space Center (Texas) which was then transferred to Kennedy Space Center (Florida) in order to be loaded into the Space Exploration Technologies (SpaceX) Dragon spacecraft prior to launch. Eight different locations were sampled on the ISS using the premoistened polyester wipes which were the same locations as those sampled during the previous microbial tracking study (MT-1). The list of locations sampled is presented in Table 1. Flight 4 sampling occurred in 2017, and flight 5, 6, and 7 sampling occurred in 2018. A diagram of locations can be found in Figure 1 of the MT-1 published manuscript [26].

As per the study requirements, there was no cleaning at least 4 days prior to sampling. Cleaning outside that time frame was conducted as per routine ISS activities and was performed with disinfectant wipes containing octyl decyl dimethyl ammonium chloride (0.0399%), dioctyl dimethyl ammonium chloride (0.01995%), didecyl dimethyl ammonium chloride (0.01995%), alkyl (50% C14, 40% C12, 10% C16) dimethylbenzylammonium chloride, and dimethylbenzylammonium chloride (0.0532%).

During each flight sampling session, the same crew member sampled each of the eight locations. Using the sterile gloves from the sampling kit, he/she sampled 1 m^2^ with the pre-moistened polyester wipes and donned a new pair of individually packed sterile gloves before sampling the next location. A control wipe (environmental control) was taken out from the zip lock bag, unfolded, waved for 30 s, and packed back inside a new sterile zip lock. One control wipe was included for each flight session. Similarly, an unused wipe that was flown to the ISS and brought back to Earth along with the samples served as a negative control for sterility testing. After sample collection, the samples were stored at 4 °C in orbit and then returned to Earth after 10 days for flight 4, 8 days for flight 5, 22 days for flight 6, and 11 days for flight 7. The kits were delivered to JPL immediately after arrival to Earth at 4 °C with processing at JPL commencing within 2 h of receipt.

### Sample processing

Sample processing took place in a cleanroom at JPL. In a certified biosafety cabinet, each wipe was aseptically removed from the zip lock bag and transferred to a 500-mL bottle containing 200 mL of molecular grade sterile phosphate-buffered saline (PBS; pH 7.4, Sigma). The wipe inside the bottle was shaken vigorously for 2 min followed by concentration with a Concentrating Pipette using 0.22-μm Hollow Fiber Polysulfone tips (Innova Prep, Drexel, MO). The captured microbes were released from the concentrating pipette using 1 mL of InnovaPrep’s elution fluid containing PBS with 0.075% Tween. The concentrate was topped up to 5 mL using sterile, molecular grade 1× PBS (pH 7.4, Sigma).

### DNA extraction

The concentrate, from above, was split into two 1.5-mL aliquots. One aliquot was treated with PMA (18.25 μL of 2 mM, resulting in a final concentration of 25 μM) to assess cells that were viable or had an intact cell membrane [51], while the second aliquot was handled in a similar manner but without the addition of PMA. The PMA- and non-PMA-treated aliquots were incubated in the dark at RT for 5 min, followed by 15 min of photoactivation using the PMA-Lite™ LED Photolysis Device, specifically designed for photoactivation of PMA (Biotium, Hayward, CA). The PMA- and non-PMA-treated aliquots were split into two 0.75-mL aliquots. One aliquot was transferred to bead beating tubes containing Lysing Matrix E (MP Biomedicals, Santa Ana, CA), followed by bead beating for 60 s using the vortex sample holder (MO Bio, Carlsbad, CA). The bead-beaten aliquot and the aliquot without bead beating were combined for their corresponding PMA-treated and non-treated samples. DNA extraction was performed with the Maxwell 16 automated system (Promega, Madison, WI), in accordance with the manufacturer’s instructions using the Maxwell 16 Tissue LEV Total RNA purification kit. A Maxwell control without any sample added in its cartridge was run concurrently with the samples. The extracted DNA was eluted in 50 μL of water and stored at − 20 °C until further analysis.

### Culture analysis

One hundred microliters, in duplicate, of the concentrate, was plated on Reasoner’s 2A agar (R2A) (for environmental bacteria), blood agar (for pathogenic bacteria), and potato dextrose agar (PDA) supplemented with 100 μg/mL of chloramphenicol (for fungi). All plates were purchased from Hardy Diagnostics (Santa Maria, CA). R2A and PDA plates were incubated at room temperature for 7 days, while blood agar plates were incubated at 37 °C for 1–2 days (depending on growth). Colonies were counted and the colony-forming units per m^2^ calculated. After colony counting, single colonies were re-streaked on fresh plates to ensure pure culture and incubated at the appropriate temperature and time, and the biomass was then archived in 30% glycerol and stored at − 80 °C.

### Shotgun metagenomics sequencing and taxonomic classification

Metagenomics sequencing and taxonomic classification were performed as described previously [30]. Briefly, DNA libraries were prepared for sequencing using the Nextera DNA Library Preparation Kit (Illumina, Inc., San Diego, CA). Quality and fragment size were assessed on the Agilent Tapestation 4200 (Agilent Technologies, Santa Clara, CA). Libraries were quantitated using the Qubit fluorimeter (Thermo Fisher Scientific, Waltham, MA) and normalized to equivalent DNA quantities, pooled, and diluted according to the manufacturer’s standard recommendations. Shotgun metagenomic sequencing was performed using an Illumina NextSeq 500 with the NextSeq Series High Output Kit v2 (Illumina Inc., San Diego, CA), using 150 base pair paired-end reads. Taxonomic classification of the metagenomic reads was accomplished using the Livermore Metagenomics Analysis Toolkit (LMAT version 1.2.4b with the “April 4, 2014, LMAT Grand” reference database [lmat-4-14.20mer.db]) [52]. Reads mapping to genus Homo or below were removed from the analysis. The raw sequencing data has been deposited in NASA’s GeneLab Database accession number GLDS-252. A list of read counts for all species detected from each sample and control wipe is shown in Dataset S4.

### Contamination control

The total number of reads and the number of reads annotated at the genus and species level are shown in Table S1. The F4–F7 negative control wipe flown to the ISS had fewer total reads compared to their corresponding flight group wipes used to sample the ISS surfaces. The microbial composition of the control samples was determined the same way as the surface samples, and the top species detected among the control samples is shown by read number (Fig. S9). The control samples showed no indication of contamination or microbial read counts that could alter the microbial profiles identified in the surface samples.

### Alpha diversity

The alpha diversity of each sample was calculated at the genus and species level using the “renyi” and “estimateR” functions available through the vegan package (v.2.5-5) [24]. The Renyi entropy of each sample was calculated for orders 0, 1, and 2, some things referred to as the Hill numbers [25], representing the sample’s observed richness, exponentiated Shannon index, and reciprocal Simpson index, respectively. We compared the alpha diversity estimations between locations and flight groups to track the changes between the sampling locations and across all flight groups. Statistical testing was done using Student’s *t*-test and Kruskal-Wallis test.

### Beta diversity and ordination

The ecological distance between the surface samples was measured using the “distance” function and visualized using the “ordinate” function from the phyloseq package (v.1.24.3) [26]. Non-metric and classical multidimensional scaling was used to visualize the similarity between the surface samples using the Jaccard and Euclidean distance, respectively. The Jaccard distance was used to measure the difference in taxon presence-absence profiles, and the Euclidean distance was used to measure the difference in taxon abundance profiles between samples. Prior to measuring the Euclidean distance between each sample, the taxon read counts were transformed into Euclidean space using the center-log ratio (clr) [27]. To determine if the centroid and dispersion between the locations and flight groups were different, a PERMANOVA test was performed using the “adonis2” function available through the vegan package (v.2.5-6) [24].

### Differential abundant analysis

Genus and species level read counts were transformed using the clr method prior to testing for differentially abundant taxa. We performed two separate differential abundance analyses. The first analysis looked at the differences in taxon abundance between the surface locations, and the second analysis looked at the differences in taxon abundance between the flight groups. Differential abundance analysis on the clr-transformed reads was performed using the ALDEx2 package (v.1.16.0) using the “aldex.kw” function [53]. Taxa with *P* values < 0.05 were considered differentially abundant, and all *P* values were adjusted for multiple testing using the Benjamini and Hochberg method [29].

### Antimicrobial resistance profiles

The raw metagenomic sequences were analyzed with DeepARG: a deep learning approach for predicting antibiotic resistance genes from metagenomic data which uses the CARD and ARDB databases for classification using the DIAMOND aligner [54]. Briefly, the pipeline first removes low-quality reads using TRIMMOMATIC, then merges the reads into one big file (VSEARCH) which are then classified with the deepARG algorithm, and the results are normalized to the 16S rRNA abundance in the sample. The parameters used to predict the AMR genes from our metagenomics dataset were based on 80% coverage, 50% homology, a minimum probability of 0.8, and an *E*-value of 1e−10.

### Co-occurrence analysis of viable organisms

Genus co-occurrence from the PMA-treated samples was examined using the “cooccur” R package. This package applied the probabilistic model of genus co-occurrence [55] to a set of genera distributed among the 32 samples (8 locations, 4 flights). The algorithm calculated the observed and expected frequencies of co-occurrence between each pair of genera. The expected frequency was based on the distribution of each genus being random and independent of the other genera. The package also includes functions for visualizing co-occurrence results and was used to prepare Figs. 8 and 9.

### Functional profiles

Metagenome assembled genomes were constructed from the 32 samples (F4–F7, non-PMA-treated samples) using the MetaWRAP pipeline (https://github.com/bxlab/metaWRAP) [56] which performed read quality control, assembly, visualization, taxonomic profiling, extracting draft genomes (binning), and functional annotation. Assembly was performed with MegaHit (due to the large dataset) and the bin_refinement cutoff set to minimum completion of 50% and maximum contamination of 10%. Taxonomy was assigned using NCBI_nt and NCBI_tax databases and verified with CheckM. The abundances of each bin (i.e., MAG) in each sample was expressed as genome copies per million reads using Salmon. Functional analysis of the annotated genomes was performed by extracting the cluster of orthologous group (COG) IDs from the annotated gff files generated by MetaWRAP and assigning each ID to a COG category (https://www.ncbi.nlm.nih.gov/research/cog/). The draft genome of *Pseudomonas granadensis* was analyzed by SEED and the Rapid Annotation of microbial genomes using Subsystems Technology (RAST) [57] and genomic islands assessed with IslandViewer 4 [58].

## Supplementary Information

**Additional file 1: Figure S1.** Microbial burden assessment using culture. Bacteria were grown on R2A and blood agar plates and the cfu/m^2^ averaged from each type of plate and then plotted. Fungi were grown on PDA plates. Each dot and corresponding number represents one of the eight surface locations sampled. X-axis labels: “BAC” refers to the bacterial load and “PDA” to the fungal load. Fx refers to one of the four flight sampling events. **Figure S2.** Alpha diversity metrics for surface samples. The species richness (top row), exponentiated Shannon index (middle row), and inverse Simpson Index (bottom row) are shown for each sample. F4 (green), F5 (yellow), F6 (purple), and F7 (red) samples are grouped by surface location. **Figure S3.** NMDS plot of MT-1 and MT-2 surface samples. Samples were colored by surface location (A) and flight group (B) to visualize samples clustering. The distance between samples was determined using the Jaccard dissimilarity. **Figure S4.** Top 12 most abundant bacterial species by flight group. The top 12 most abundance fungal species for F4 (A), F5 (B), F6 (C), and F7 (D) were determined separately. The read counts for each species are shown by surface location. “Other” refers to those bacterial species detected that were not in the top 12. **Figure S5.** Top 12 most abundant fungal species by flight group. The top 12 most abundance fungal species for F4 (A), F5 (B), F6 (C), and F7 (D) were determined separately. The read counts for each species are shown by surface location. “Other” refers to those fungal species detected that were not in the top 12. **Figure S6.** Co-occurrence analysis. Co-occurrence analysis was performed using the 50 most abundant genera to determine associations amongst organisms. The x-axis shows the organisms that were included in the analysis and the y-axis shows the percent of either negative, positive or random associations. Gray bars represent random associations, blue bars represent positive associations and yellow bars represent negative associations. The genera are listed in order of least occurrences (left) to most occurrences (right). **Figure S7.** Heat map of ISS resistome. The metagenomic dataset was analyzed with DeepARG to look for anti-microbial resistance genes. The genes were then grouped into classes of resistance (i.e “beta-lactam”), which is displayed in this heatmap (y-axis). The heat-map shows the relative abundance of each resistance class in each sample collected, with red being the most abundant and green the least. Gray indicates zero counts detected in that sample The samples analyzed (x-axis) are from the non-PMA treated samples. x-axis naming: Fx_xS, with F referring to the flight (i.e., F4) and S referring to the location of the surface sampled (i.e., 1S is location 1, the port panel next to cupola). The AMR counts were normalized based on the 16S rRNA counts. **Figure S8.** Subsystem category distribution of *Pseudomonas fluroescens* metagenome assembled genome. The metaWRAP pipeline was used to assemble and annotate draft genomes constructed from the metagenomic data of 32 samples (F4-F7), otherwise known as MAGs- metagenomic assembled genomes. The MAG of *Pseudomonas fluorescens* was further analyzed with SEED and RAST. **Figure S9.** Read counts for control samples. The top 12 most abundance species across the control samples are colored. The read counts in the controls were significantly less than those detected in the samples and had different profiles.

**Additional file 2: Table S1.** List of reads obtained from sample and control wipes during each ISS flight.

**Additional file 3: Dataset S1.** Read counts by Kingdom.

**Additional file 4: Dataset S2.** The percent completion, contamination, GC content, taxonomy, N50 (a measure of assembly quality) and genome size.

**Additional file 5: Dataset S3.** Complete list of each COG ID.

**Additional file 6: Dataset S4.** Read counts of detected species in each sample and control. Only those species with over 100 read counts across all samples/controls were included. “F” referes to flight group, xS referes to location. “P” refers to PMA treatment, “CTL” refers to control wipe fliown to ISS.

## Data Availability

The raw sequencing data has been deposited in NASA’s GeneLab Database accession number GLDS-252 (https://genelab-data.ndc.nasa.gov/genelab/accession/GLDS-252/) and NCBI SRA under accession number PRJNA781277 (https://www.ncbi.nlm.nih.gov/search/all/?term=PRJNA781277).
