# Supplementary material for: Microbial Tracking-2, a metagenomics analysis of bacteria and fungi onboard the International Space Station
Source: Microbiome. 2022 Jun 29;10:100. doi: 10.1186/s40168-022-01293-0 (PMC9241228; doi:10.1186/s40168-022-01293-0)
Supplement: Supplementary file 2 — Additional file 1: Figure S1. Microbial burden assessment using culture. Bacteria were grown on R2A and blood agar plates and the cfu/m2 averaged from each type of plate and then plotted. Fungi were grown on PDA plates. Each dot and corresponding number represents one of the eight surface locations sampled. X-axis labels: “BAC” refers to the bacterial load and “PDA” to the fungal load. Fx refers to one of the four flight sampling events. Figure S2. Alpha diversity metrics for surface samples. The species richness (top row), exponentiated Shannon index (middle row), and inverse Simpson Index (bottom row) are shown for each sample. F4 (green), F5 (yellow), F6 (purple), and F7 (red) samples are grouped by surface location. Figure S3. NMDS plot of MT-1 and MT-2 surface samples. Samples were colored by surface location (A) and flight group (B) to visualize samples clustering. The distance between samples was determined using the Jaccard dissimilarity. Figure S4. Top 12 most abundant bacterial species by flight group. The top 12 most abundance fungal species for F4 (A), F5 (B), F6 (C), and F7 (D) were determined separately. The read counts for each species are shown by surface location. “Other” refers to those bacterial species detected that were not in the top 12. Figure S5. Top 12 most abundant fungal species by flight group. The top 12 most abundance fungal species for F4 (A), F5 (B), F6 (C), and F7 (D) were determined separately. The read counts for each species are shown by surface location. “Other” refers to those fungal species detected that were not in the top 12. Figure S6. Co-occurrence analysis. Co-occurrence analysis was performed using the 50 most abundant genera to determine associations amongst organisms. The x-axis shows the organisms that were included in the analysis and the y-axis shows the percent of either negative, positive or random associations. Gray bars represent random associations, blue bars represent positive associations and yellow bars repre [file 40168_2022_1293_MOESM1_ESM.pdf]

Fig. S1

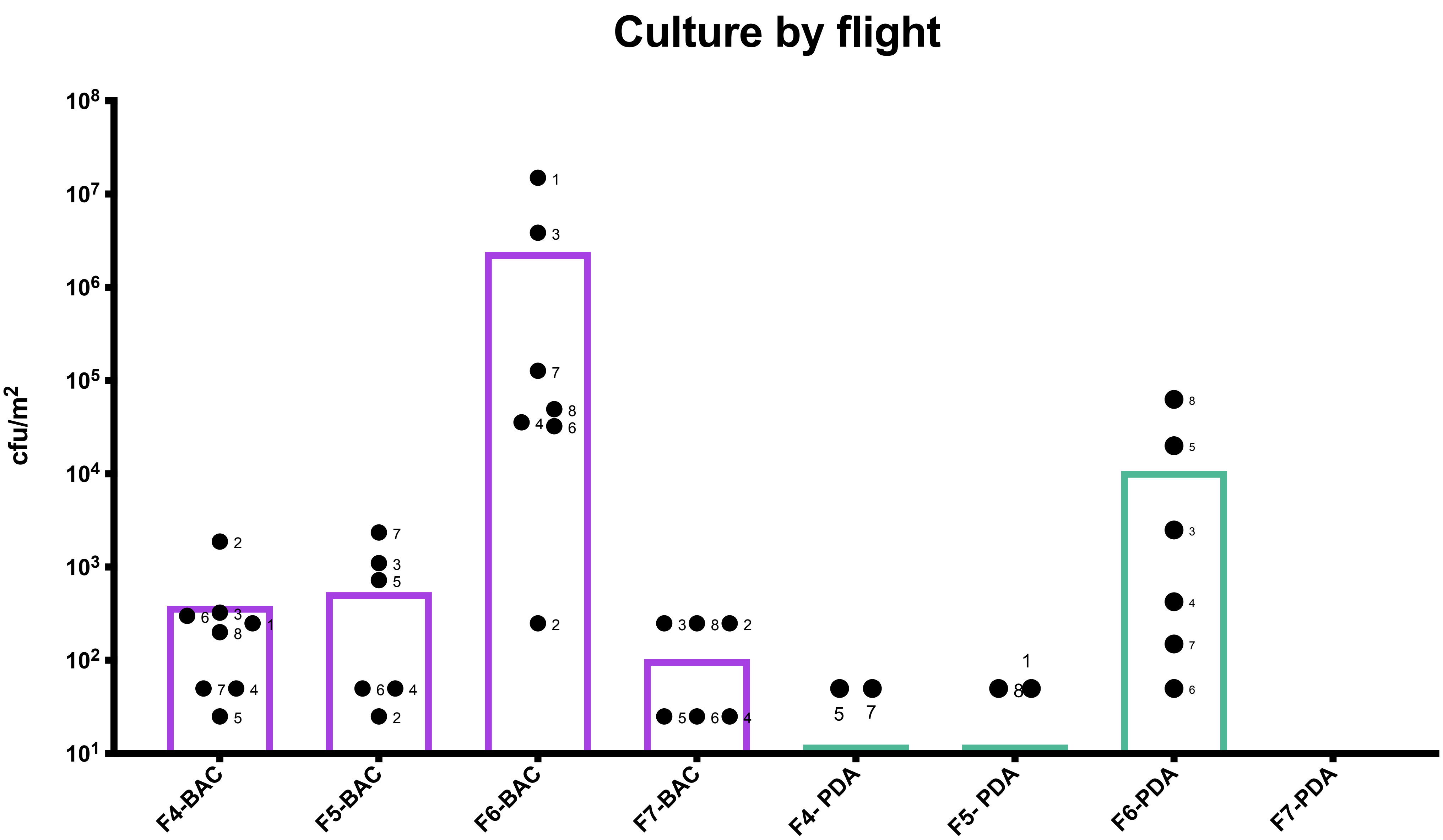

Culture by flight
cfu/m2
10^8
10^7
10^6
10^5
10^4
10^3
10^2
10^1
F4-BAC
F5-BAC
F6-BAC
F7-BAC
F4- PDA
F5- PDA
F6-PDA
F7-PDA

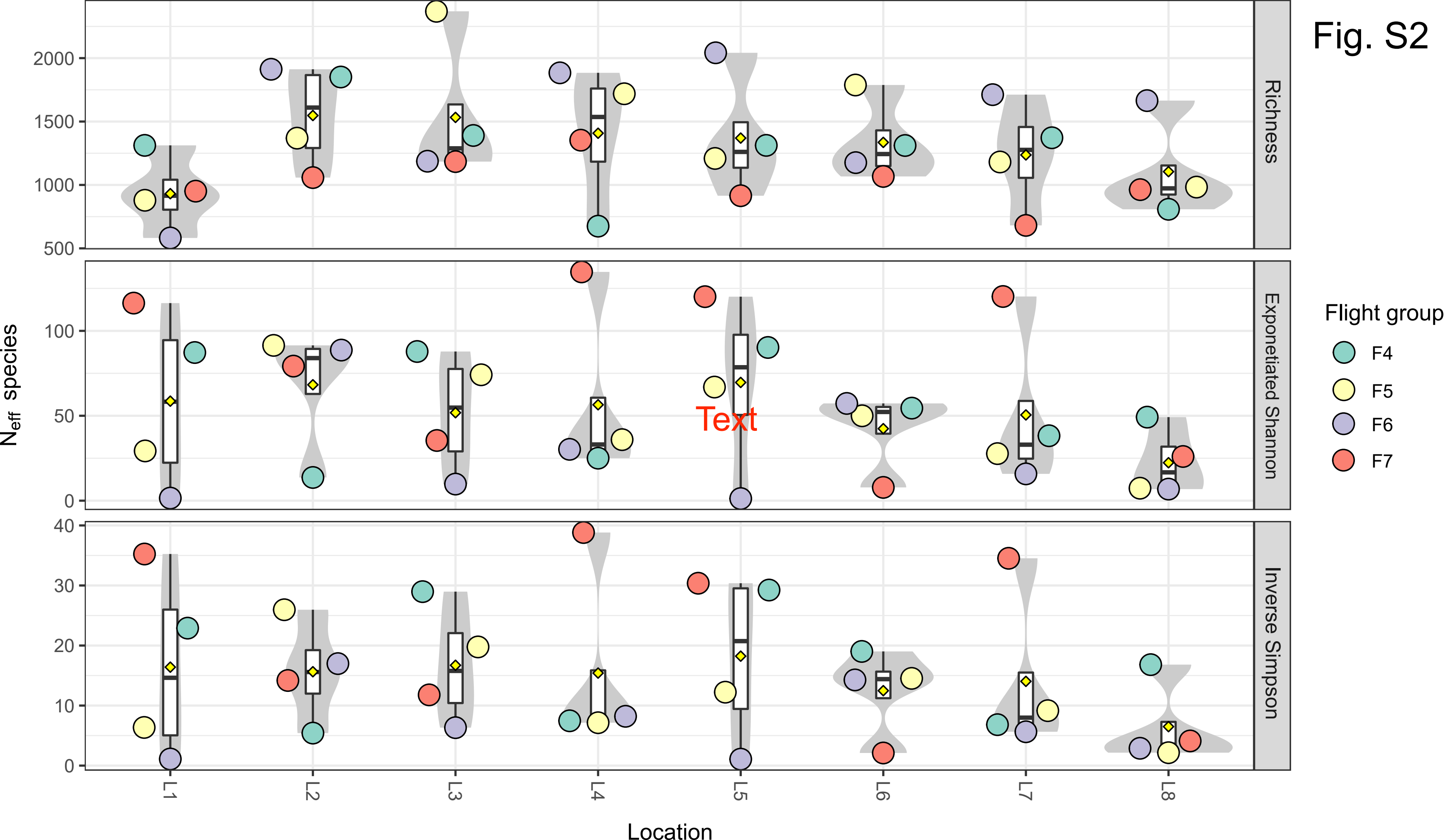
Fig. S2
Richness
Exponetiated Shannon
Inverse Simpson
N_{eff} species
Location
L1
L2
L3
L4
L5
L6
L7
L8
2000
1500
1000
500
100
50
0
40
30
20
10
Text
Flight group
F4
F5
F6
F7

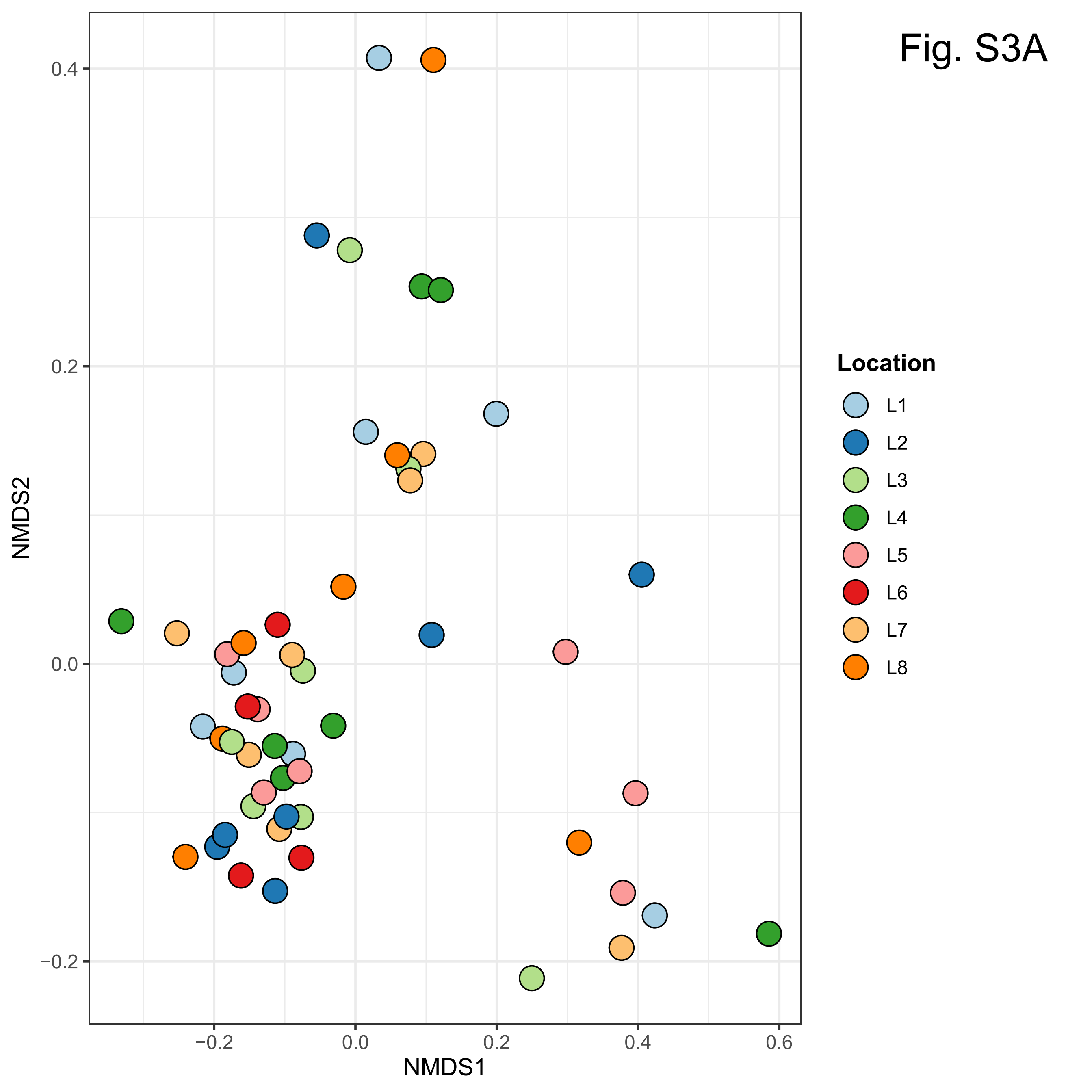
Fig. S3A
NMDS2
NMDS1
0.4
0.2
0.0
−0.2
−0.2
0.0
0.2
0.4
0.6
Location
L1
L2
L3
L4
L5
L6
L7
L8

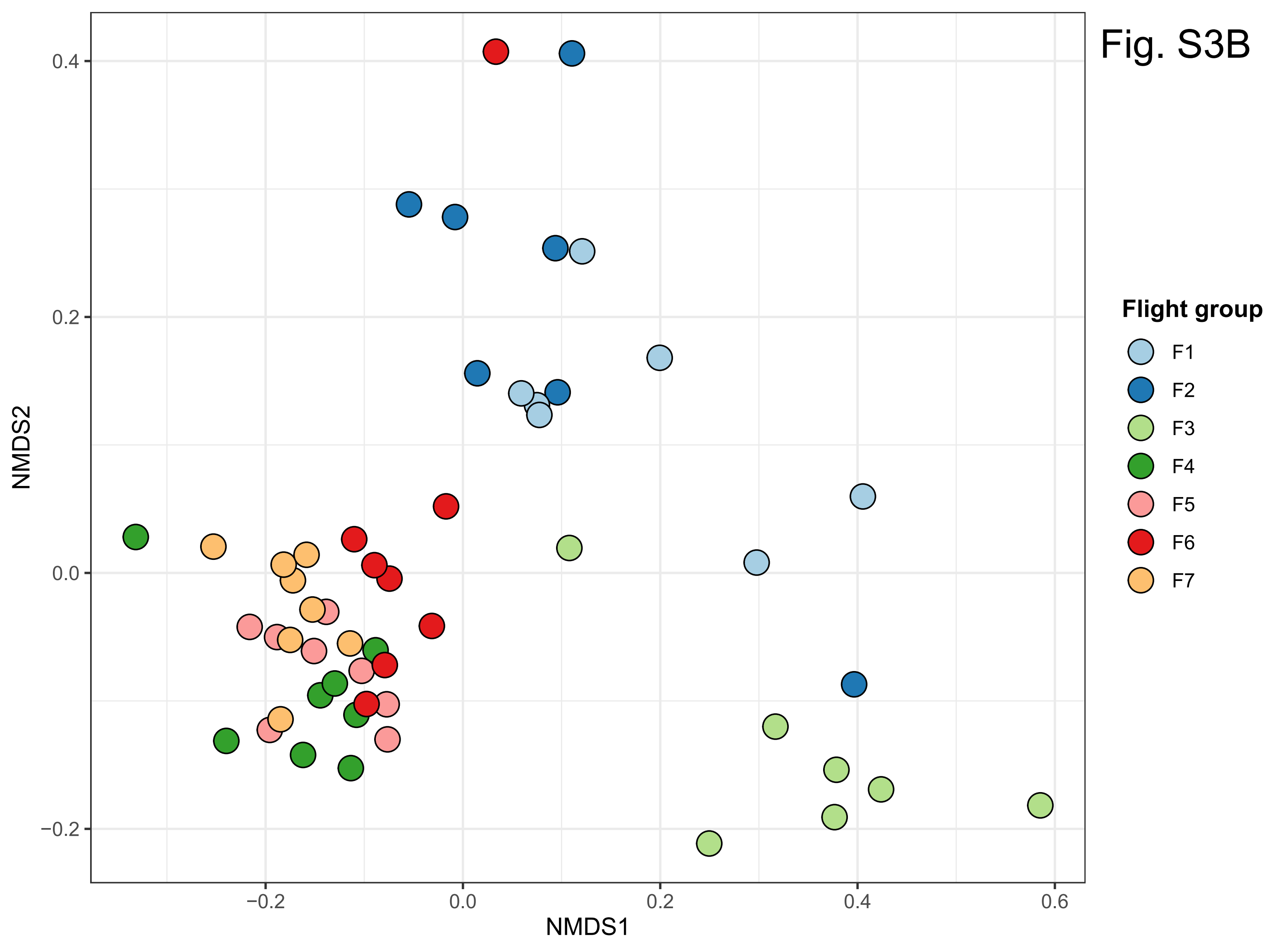

Fig. S3B
Flight group
F1
F2
F3
F4
F5
F6
F7
NMDS1
NMDS2
0.4
0.2
0.0
−0.2
−0.2
0.0
0.2
0.4
0.6

Fig. S4

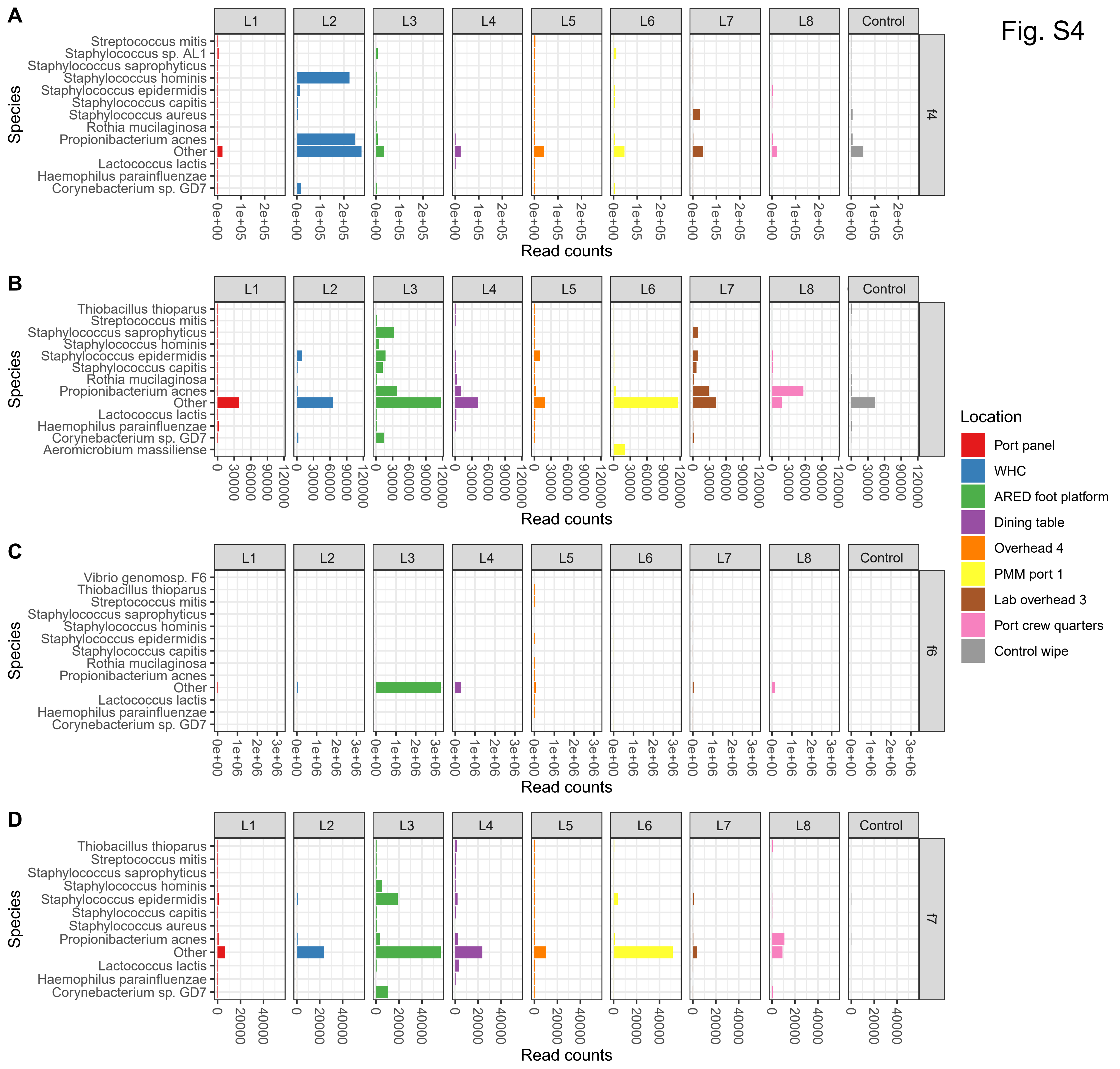
A
B
C
D
L1
L2
L3
L4
L5
L6
L7
L8
Control
f4
f6
f7
Species
Read counts
Vibrio genomosp. F6
Thiobacillus thioparus
Streptococcus mitis
Staphylococcus sp. AL1
Staphylococcus saprophyticus
Staphylococcus hominis
Staphylococcus epidermidis
Staphylococcus capitis
Staphylococcus aureus
Rothia mucilaginosa
Propionibacterium acnes
Other
Lactococcus lactis
Haemophilus parainfluenzae
Corynebacterium sp. GD7
Aeromicrobium massiliense
0e+00
1e+05
2e+05
0
30000
60000
90000
120000
1e+06
2e+06
3e+06
20000
40000
Location
Port panel
WHC
ARED foot platform
Dining table
Overhead 4
PMM port 1
Lab overhead 3
Port crew quarters
Control wipe

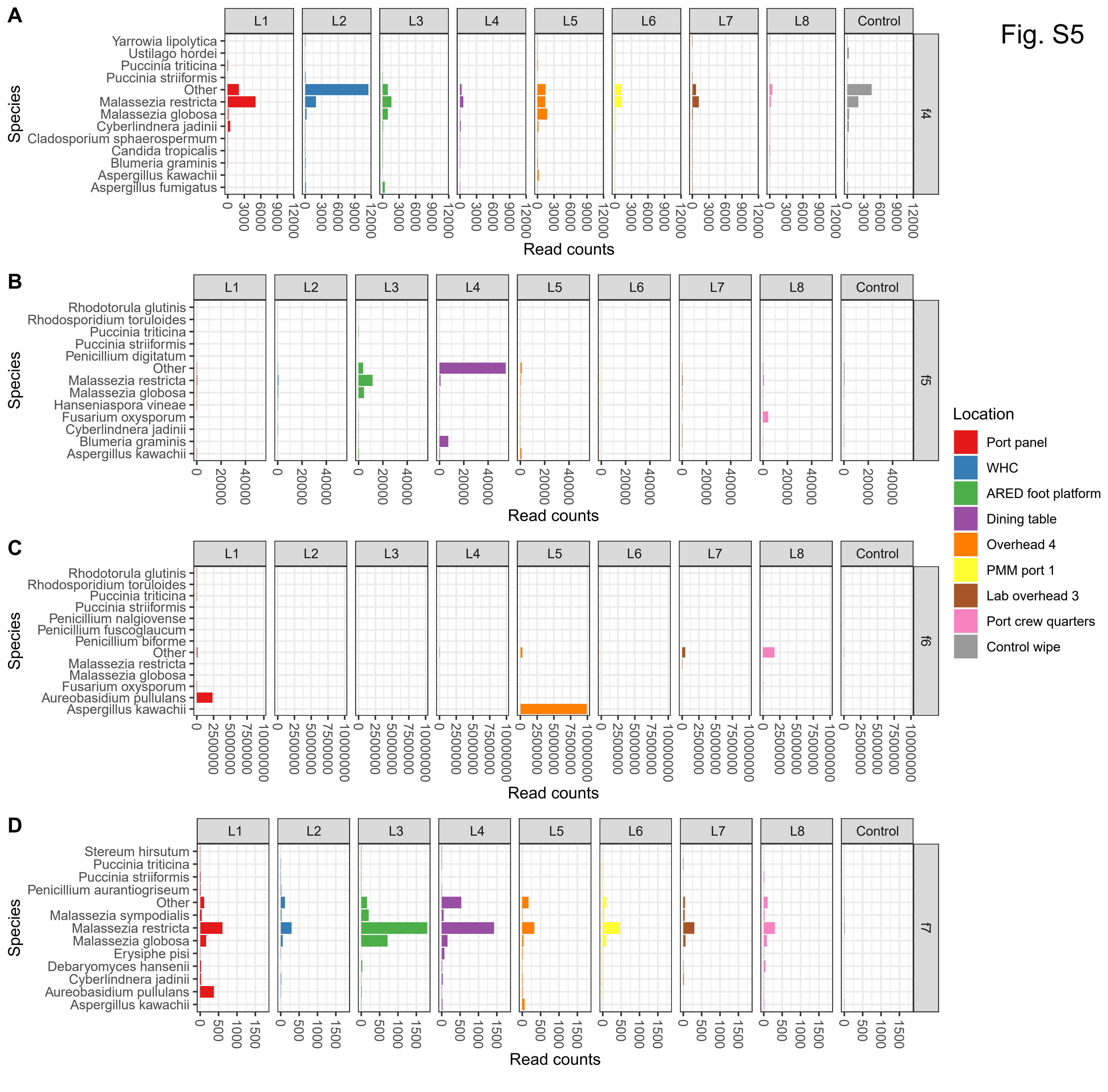
Fig. S5
A
B
C
D
L1
L2
L3
L4
L5
L6
L7
L8
Control
f4
f5
f6
f7
Species
Read counts
Yarrowia lipolytica
Ustilago hordei
Puccinia triticina
Puccinia striiformis
Other
Malassezia restricta
Malassezia globosa
Cyberlindnera jadinii
Cladosporium sphaerospermum
Candida tropicalis
Blumeria graminis
Aspergillus kawachii
Aspergillus fumigatus
Rhodotorula glutinis
Rhodosporidium toruloides
Penicillium digitatum
Hanseniaspora vineae
Fusarium oxysporum
Penicillium nalgiovense
Penicillium fuscoglaucum
Penicillium biforme
Aureobasidium pullulans
Stereum hirsutum
Penicillium aurantiogriseum
Malassezia sympodialis
Erysiphe pisi
Debaryomyces hansenii
Location
Port panel
WHC
ARED foot platform
Dining table
Overhead 4
PMM port 1
Lab overhead 3
Port crew quarters
Control wipe

Fig. S6

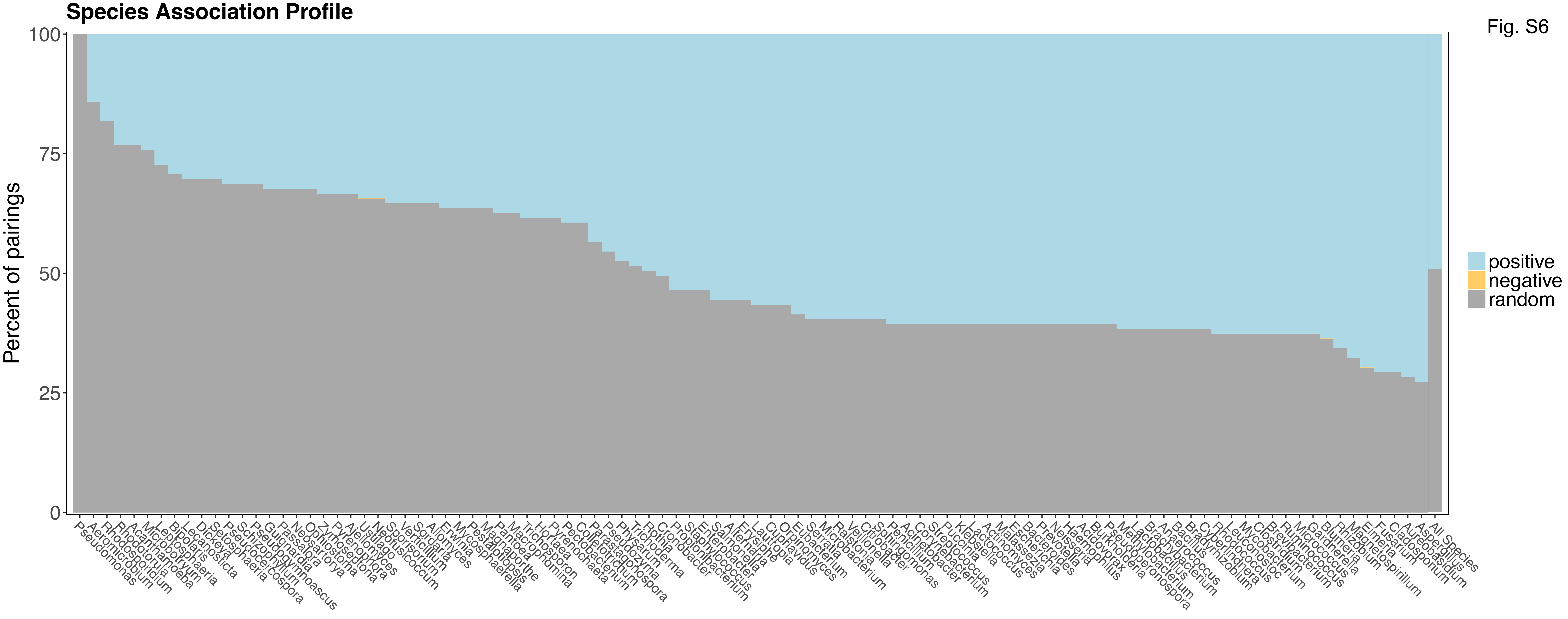

Species Association Profile
Percent of pairings
100
75
50
25
0
positive
negative
random
Pseudomonas
All Species

Fig. S7

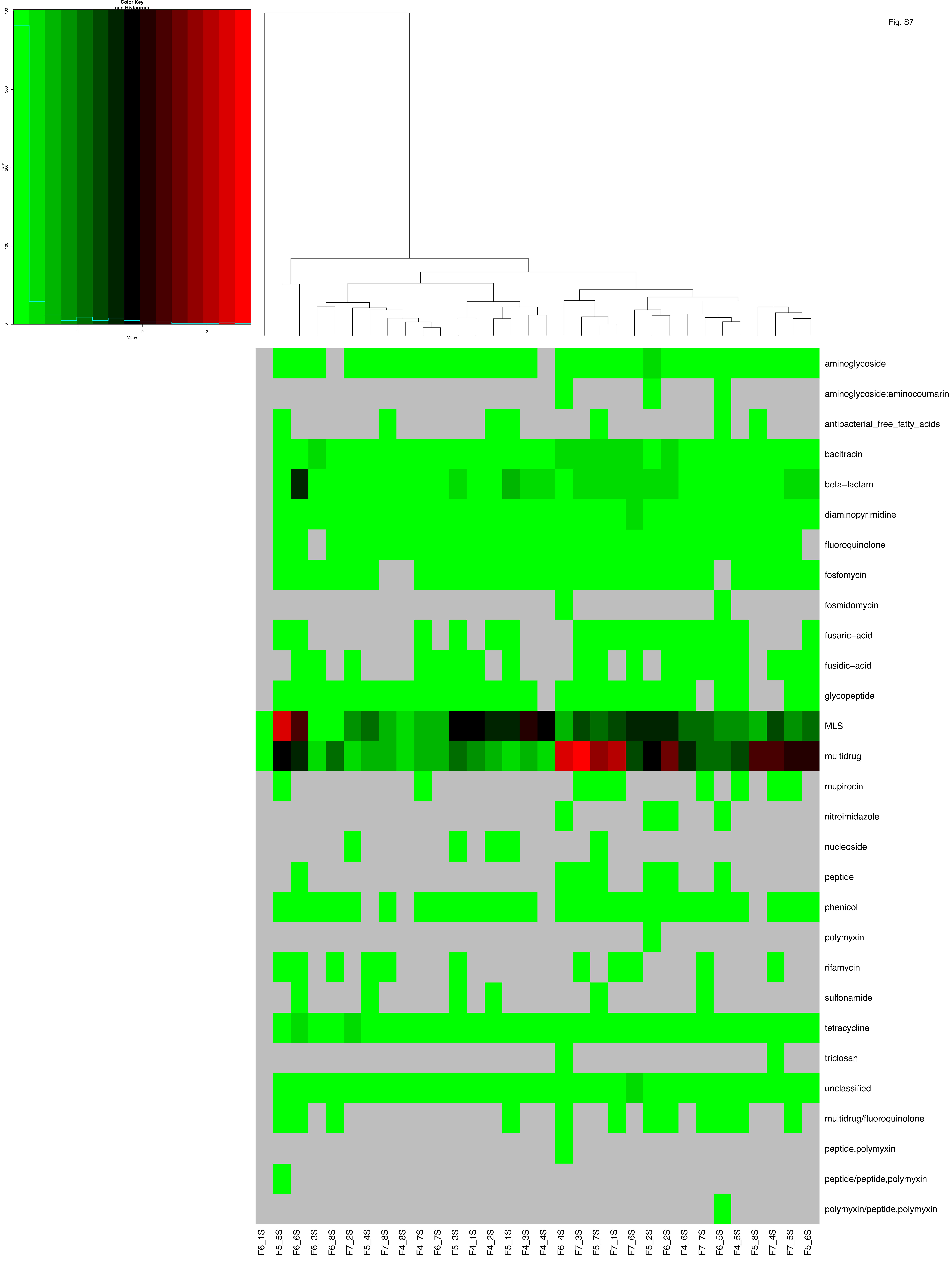
Color Key
and Histogram
Count
Value
0
100
200
300
400
1
2
3
aminoglycoside
aminoglycoside:aminocoumarin
antibacterial_free_fatty_acids
bacitracin
beta−lactam
diaminopyrimidine
fluoroquinolone
fosfomycin
fosmidomycin
fusaric−acid
fusidic−acid
glycopeptide
MLS
multidrug
mupirocin
nitroimidazole
nucleoside
peptide
phenicol
polymyxin
rifamycin
sulfonamide
tetracycline
triclosan
unclassified
multidrug/fluoroquinolone
peptide,polymyxin
peptide/peptide,polymyxin
polymyxin/peptide,polymyxin
F6_1S
F5_5S
F6_6S
F6_3S
F6_8S
F7_2S
F5_4S
F7_8S
F4_8S
F4_7S
F6_7S
F5_3S
F4_1S
F4_2S
F5_1S
F4_3S
F4_4S
F6_4S
F7_3S
F5_7S
F7_1S
F7_6S
F5_2S
F6_2S
F4_6S
F7_7S
F6_5S
F4_5S
F5_8S
F7_4S
F7_5S
F5_6S

Fig. S8

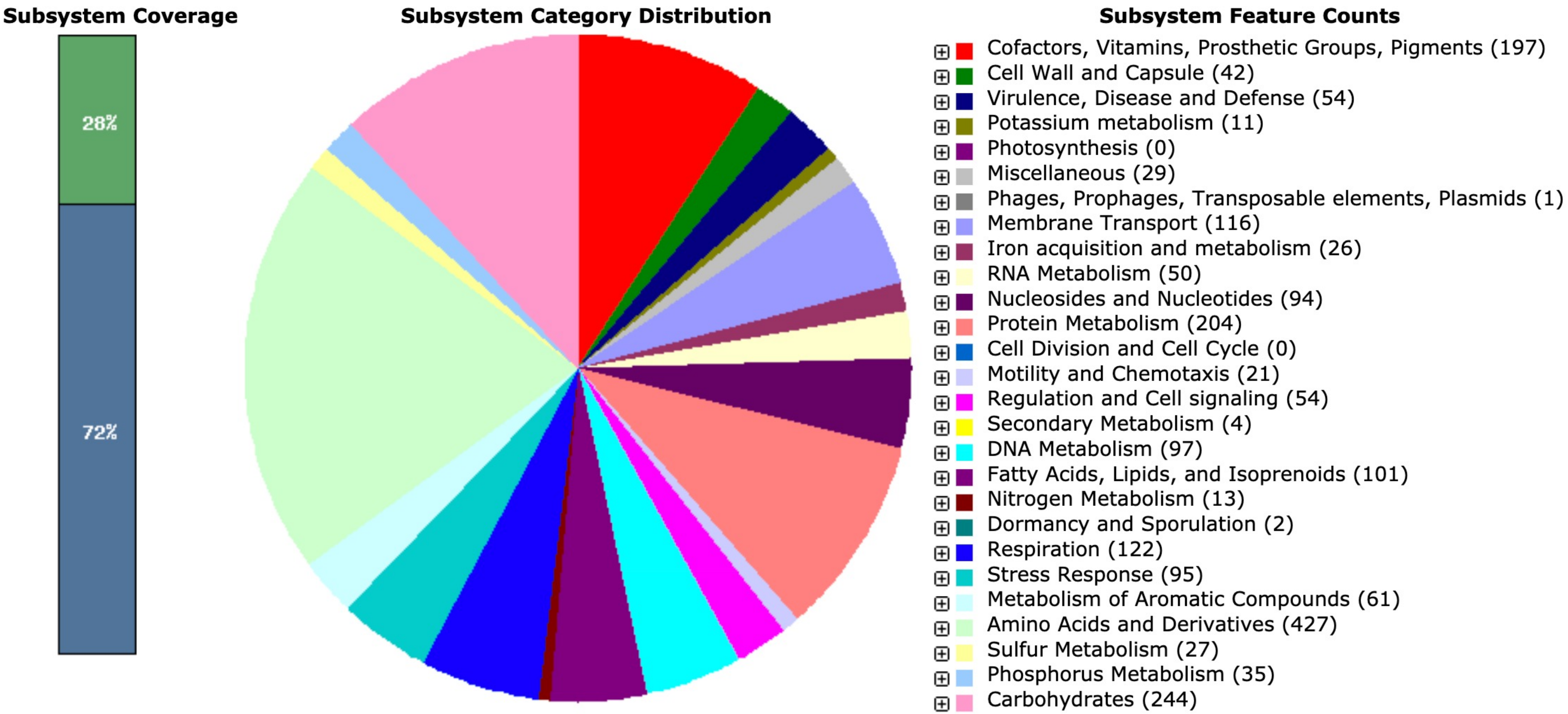
Subsystem Coverage
28%
72%
Subsystem Category Distribution
Subsystem Feature Counts
Cofactors, Vitamins, Prosthetic Groups, Pigments (197)
Cell Wall and Capsule (42)
Virulence, Disease and Defense (54)
Potassium metabolism (11)
Photosynthesis (0)
Miscellaneous (29)
Phages, Prophages, Transposable elements, Plasmids (1)
Membrane Transport (116)
Iron acquisition and metabolism (26)
RNA Metabolism (50)
Nucleosides and Nucleotides (94)
Protein Metabolism (204)
Cell Division and Cell Cycle (0)
Motility and Chemotaxis (21)
Regulation and Cell signaling (54)
Secondary Metabolism (4)
DNA Metabolism (97)
Fatty Acids, Lipids, and Isoprenoids (101)
Nitrogen Metabolism (13)
Dormancy and Sporulation (2)
Respiration (122)
Stress Response (95)
Metabolism of Aromatic Compounds (61)
Amino Acids and Derivatives (427)
Sulfur Metabolism (27)
Phosphorus Metabolism (35)
Carbohydrates (244)

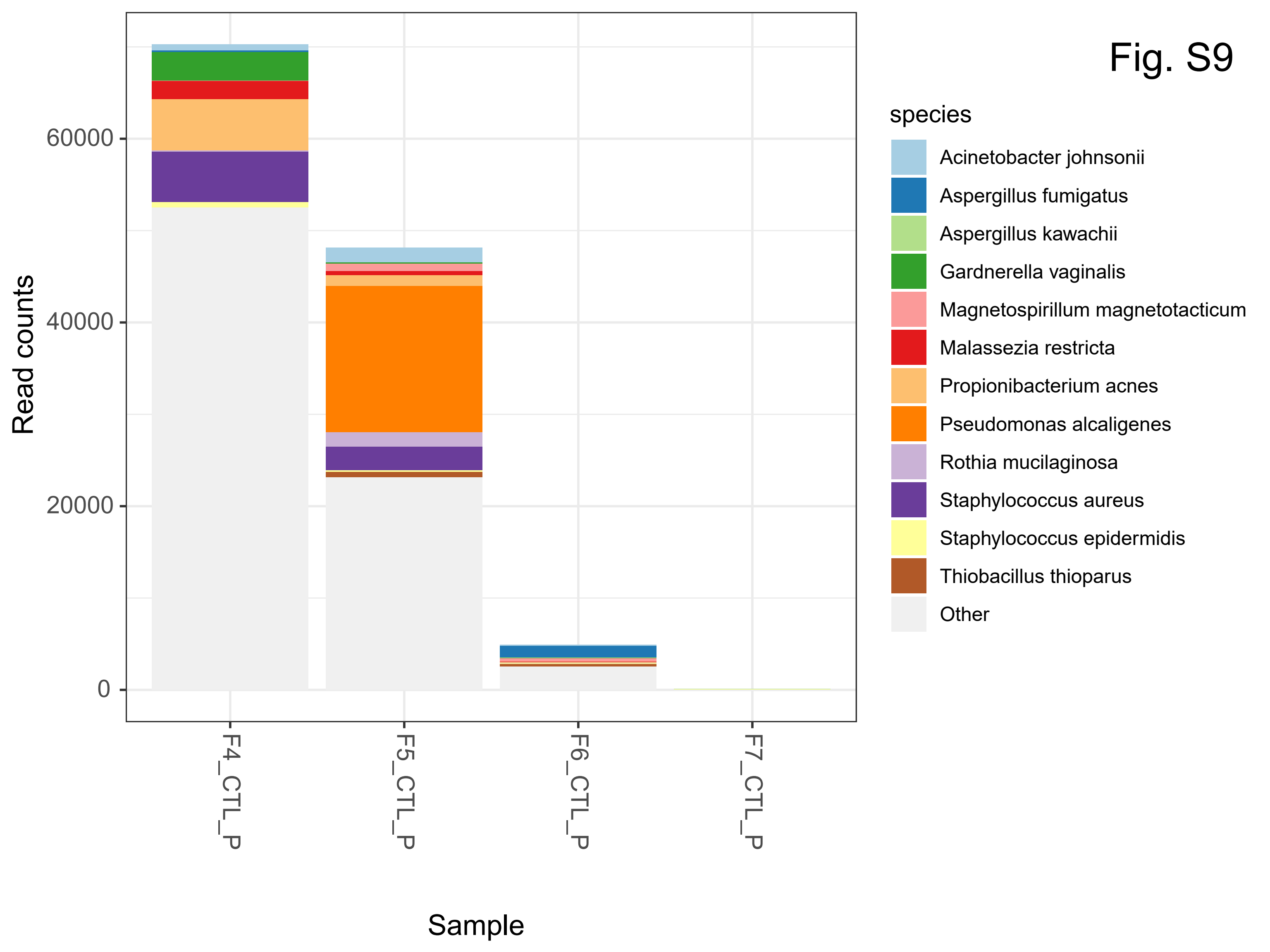

Fig. S9
Read counts
60000
40000
20000
0
Sample
F4_CTL_P
F5_CTL_P
F6_CTL_P
F7_CTL_P
species
Acinetobacter johnsonii
Aspergillus fumigatus
Aspergillus kawachii
Gardnerella vaginalis
Magnetospirillum magnetotacticum
Malassezia restricta
Propionibacterium acnes
Pseudomonas alcaligenes
Rothia mucilaginosa
Staphylococcus aureus
Staphylococcus epidermidis
Thiobacillus thioparus
Other
