# Supplementary material for: Microbial Tracking-2, a metagenomics analysis of bacteria and fungi onboard the International Space Station
Source: Microbiome. 2022 Jun 29;10:100. doi: 10.1186/s40168-022-01293-0 (PMC9241228; doi:10.1186/s40168-022-01293-0)
Supplement: Supplementary file 3 — Additional file 2: Table S1. List of reads obtained from sample and control wipes during each ISS flight. [file 40168_2022_1293_MOESM2_ESM.docx]

**Table S1**: List of reads obtained from sample and control wipes during each ISS flight.

| **Sample** | **Sample type** | **Flight group** | **Total number of reads** | **Number of reads annotated at genus level** | **Number of reads annotated at species level** |
| --- | --- | --- | --- | --- | --- |
| F4_1S_P | sample | f4 | 135762 | 93404 | 38365 |
| F4_2S_P | sample | f4 | 15815008 | 15474165 | 803846 |
| F4_3S_P | sample | f4 | 2040976 | 364844 | 68903 |
| F4_4S_P | sample | f4 | 51608 | 44653 | 26653 |
| F4_5S_P | sample | f4 | 240271 | 155318 | 59689 |
| F4_6S_P | sample | f4 | 567289 | 504757 | 84501 |
| F4_7S_P | sample | f4 | 631289 | 588905 | 79875 |
| F4_8S_P | sample | f4 | 280281 | 124696 | 28386 |
| F5_1S_P | sample | f5 | 107409 | 93060 | 44393 |
| F5_2S_P | sample | f5 | 257255 | 159127 | 84094 |
| F5_3S_P | sample | f5 | 947738 | 637019 | 260696 |
| F5_4S_P | sample | f5 | 343743 | 243534 | 125187 |
| F5_5S_P | sample | f5 | 225021 | 180749 | 39435 |
| F5_6S_P | sample | f5 | 287005 | 215759 | 146625 |
| F5_7S_P | sample | f5 | 384584 | 329637 | 100132 |
| F5_8S_P | sample | f5 | 338516 | 255474 | 83600 |
| F6_1S_P | sample | f6 | 3407386 | 2486554 | 2466483 |
| F6_2S_P | sample | f6 | 331641 | 185427 | 95259 |
| F6_3S_P | sample | f6 | 14553392 | 13914605 | 3261988 |
| F6_4S_P | sample | f6 | 4620420 | 1893912 | 320690 |
| F6_5S_P | sample | f6 | 20389698 | 19149693 | 10243718 |
| F6_6S_P | sample | f6 | 102261 | 63088 | 37252 |
| F6_7S_P | sample | f6 | 2002671 | 878601 | 541114 |
| F6_8S_P | sample | f6 | 24677213 | 21818519 | 1900387 |
| F7_1S_P | sample | f7 | 50245 | 36934 | 11867 |
| F7_2S_P | sample | f7 | 74577 | 47936 | 26647 |
| F7_3S_P | sample | f7 | 383533 | 334986 | 99962 |
| F7_4S_P | sample | f7 | 1344344 | 121664 | 38386 |
| F7_5S_P | sample | f7 | 61421 | 30412 | 12896 |
| F7_6S_P | sample | f7 | 154183 | 110593 | 58824 |
| F7_7S_P | sample | f7 | 31062 | 21571 | 6223 |
| F7_8S_P | sample | f7 | 67405 | 50301 | 22268 |
| F4_CTL_P | control | f4 | 209046 | 173581 | 70277 |
| F5_CTL_P | control | f5 | 98325 | 80304 | 48138 |
| F6_CTL_P | control | f6 | 17681 | 9915 | 4932 |
| F7_CTL_P | control | f7 | 419 | 278 | 79 |
